# Histone lactylation in fibroblast-like synoviocytes drives CXCL1-mediated immune cell recruitment in rheumatoid arthritis

**DOI:** 10.1016/j.isci.2026.118108

**Published:** 2026-11-04

**Authors:** Zhidong Liu, Xiaojun Xu, Chaoqiang Chen, Zhexiao Lan, Peitao Xu, Jinteng Li, Guiwen Ye

**Affiliations:** 1 Department of Orthopedics, The Eighth Affiliated Hospital of Sun Yat-Sen University, Shenzhen, China

**Keywords:** rheumatoid arthritis, fibroblast-like synoviocytes, histone lactylation, CXCL1, immune cell recruitment

## Abstract

Activated fibroblast-like synoviocytes (FLS) drive rheumatoid arthritis (RA) progression by recruiting immune cells. Metabolic reprogramming, marked by enhanced glycolysis and lactate accumulation, contributes to RA pathogenesis. Here, an analysis of a public metabolomics dataset identified elevated plasma lactate in patients with RA. Histone lactylation was increased in synovial FLS from patients with RA and was linked to upregulated expression of the chemokine CXCL1 and enhanced immune cell recruitment. In collagen-induced arthritis and SKG mouse models, prophylactic CXCL1 neutralization or lactate dehydrogenase inhibition reduced immune cell infiltration, alleviated joint inflammation, and mitigated bone destruction. Lactate dehydrogenase inhibition also reduced synovial lactate levels, histone lactylation, and CXCL1 expression. These findings support a role for histone lactylation in FLS-mediated immune cell recruitment and suggest that this pathway warrants further investigation as a therapeutic target in RA.

## Highlights

• Histone lactylation is upregulated in FLS in rheumatoid arthritis
• Histone lactylation in FLS promotes CXCL1 secretion and immune recruitment
• Targeting histone lactylation or CXCL1 reduces inflammation and bone destruction
• LDHA inhibitor stiripentol targets histone lactylation to mitigate rheumatoid arthritis

## Graphical abstract

**Figure:**
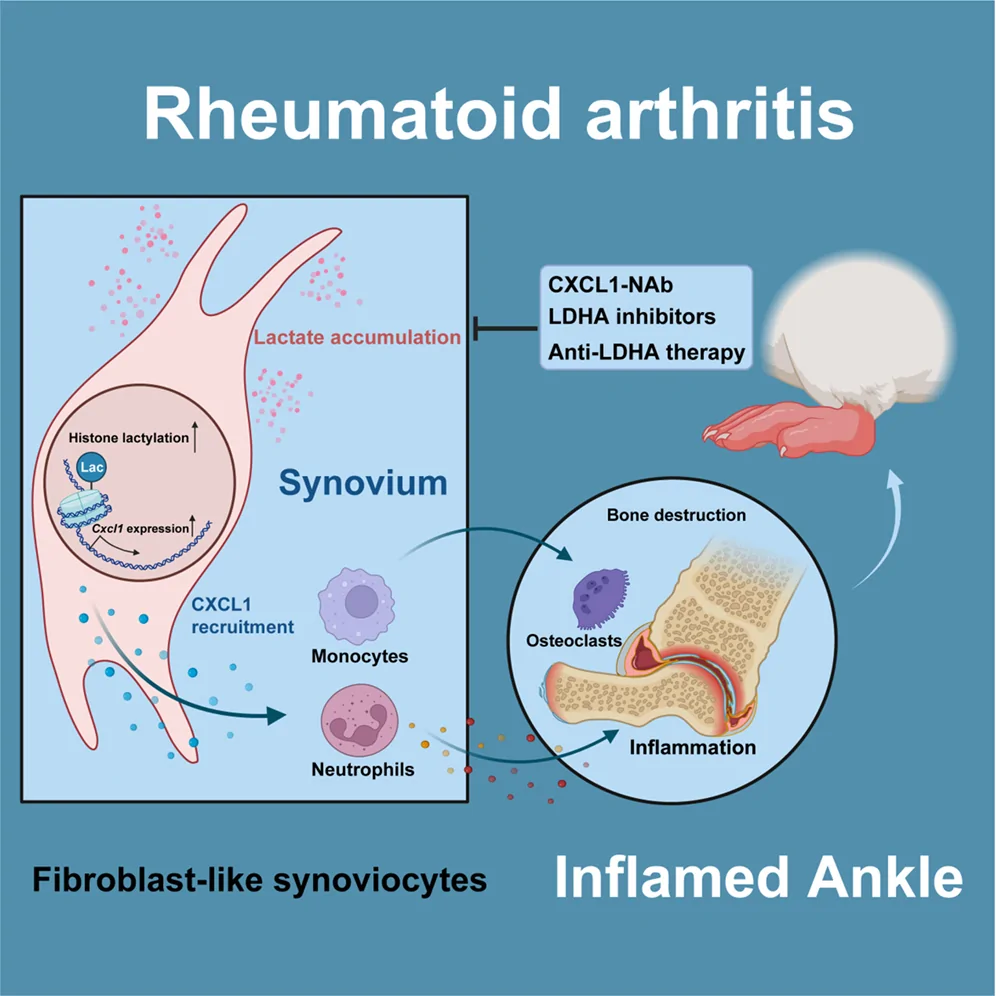

## Teaser

Molecular biology; Immunology; Cell biology

## Introduction

Autoimmune arthritis, including rheumatoid arthritis (RA), ankylosing spondylitis (AS), psoriatic arthritis (PsA), systemic lupus erythematosus (SLE) arthritis, and pediatric arthritis, encompasses a group of autoimmune diseases characterized by progressive bone and joint destruction.^1^^,^^2^^,^^3^^,^^4^ Despite differences in their core pathogenesis, excessive recruitment of immune cells is a universal pathological feature that plays a central role in disease progression.^5^ Therefore, targeting immune cell recruitment represents a promising direction for novel therapeutic strategies for autoimmune arthritis.^6^ Among these, RA is the most prevalent and extensively studied prototype, in which immune cell dysregulation and joint erosion are particularly well characterized.

Fibroblast-like synoviocytes (FLS), the principal mesenchymal stromal cells in the synovium, play a critical role in maintaining joint architecture.^7^ Accumulating evidence indicates that FLS actively drive RA pathology by acting as local pro-inflammatory mediators.^8^ This occurs primarily through chemokine secretion, which recruits immune cells, although the underlying mechanisms remain poorly understood.^9^ Suppressing the aberrant recruitment activity of FLS reduces inflammatory cell infiltration in affected joints and attenuates arthritis symptoms.^10^ Importantly, because FLS are non-immune cells, targeting them may carry a lower risk of compromising systemic immune function, potentially reducing adverse effects associated with systemic immunosuppression.^10^ Elucidating the mechanisms governing the chemotactic phenotype of FLS may enable the development of more effective therapies with fewer systemic side effects for RA.

Similar to the Warburg effect observed in tumors, aerobic glycolysis is a hallmark of RA and plays a critical role in disease progression through multiple mechanisms.^11^ Hyperactive aerobic glycolysis has been reported in FLS within arthritic lesions, but its contribution to the recruitment phenotype of FLS remains unclear.^12^ Lactate, the glycolytic end-product, accumulates in RA lesions owing to overactive glycolysis.^13^ Once considered merely a metabolic by-product, lactate is now recognized as a key regulator of diverse pathological processes. Notably, Zhang et al. discovered that lactate drives a novel post-translational modification (PTM)—termed lactylation—that serves as an epigenetic regulator of gene transcription.^14^ Subsequent studies have established that histone lactylation plays critical roles in both physiological and pathological processes.^15^^,^^16^ However, whether histone lactylation contributes to RA pathogenesis or mediates the recruitment phenotype of FLS in arthritic lesions remains largely unexplored.

In this study, an analysis of a publicly available metabolomics dataset identified elevated plasma lactate as a metabolite associated with RA. We further showed that lactate-mediated histone lactylation is significantly elevated in FLS from RA lesions and positively regulates their recruitment phenotype by promoting Cxcl1 transcription. *In vivo*, neutralizing CXCL1 or inhibiting histone lactylation markedly attenuated arthritis severity and bone destruction by reducing inflammatory cell infiltration. Together, our findings suggest that targeting histone lactylation to block the recruitment phenotype of FLS represents a promising therapeutic strategy for RA.

## Results

### Elevated levels of lactate and histone lactylation represent clinically relevant features associated with RA

To investigate the clinical relevance of lactate in RA, we developed a metabolomics-based prediction model integrating least absolute shrinkage and selection operator (LASSO) regression and random forest algorithms using serum metabolomics data from healthy controls and patients with RA (Figures 1A and 1B). To prevent data leakage, LASSO feature selection was performed exclusively within the training set, while the test set was held out until final model evaluation. We employed 5-fold cross-validation to identify the optimal log lambda regularization parameter (Figure 1C). Using this lambda value, 66 metabolites with strong associations were selected from a total of 302 (Figure 1D). Subsequently, these 66 metabolites were used to construct a predictive model for diagnosing RA based on serum metabolomic profiles. The number of trees in the random forest was then optimized to minimize the model’s prediction error (Figure 1E). The model demonstrated exceptional predictive performance, achieving an area under the receiver operating characteristic (ROC) curve (AUC) of 0.9891 (Figure 1F). To further assess robustness, we performed nested cross-validation (outer 5-fold × inner 5-fold), yielding a bias-corrected AUC of 0.9889 ± 0.0222, confirming the stability of the multi-metabolite model. Notably, variable importance analysis through the Gini importance revealed DL-lactate as a critical contributor to model efficacy, highlighting its significant role in the diagnosis of RA (Figure 1G). When evaluated as a single metabolite, DL-lactate yielded a test-set AUC of 0.7880 and a 5-fold cross-validated AUC of 0.8961 ± 0.0881, with a Brier score of 0.0555 and well-calibrated probability predictions (calibration curve in Figure 1F). These results indicate that, within this discovery cohort, lactate is associated with RA, though its standalone discriminative ability is moderate. Beyond its classical role as a glycolytic end product, lactate has emerged as a substrate for histone lactylation, a novel epigenetic modification that potentially regulates gene expression in autoimmune pathology. FLS play a critical role in RA pathogenesis by driving synovial inflammation.^17^ To investigate histone lactylation levels in FLS from patients with RA, synovial tissues were collected from patients undergoing surgery who had RA and osteoarthritis (OA). Given the relatively mild synovial inflammation in patients with OA, these samples served as controls.^18^ Immunofluorescence staining of synovial tissue sections revealed significantly elevated levels of histone pan-lysine lactylation (Pan-Kla) in RA-derived FLS compared to OA-derived FLS, using PDGFR-α as an FLS marker^19^ (Figure 1H). Furthermore, established histone lactylation sites implicated in various diseases—specifically H3K9la, H3K18la, H4K8la, and H4K12la—were consistently upregulated in RA-derived FLS, as confirmed by immunofluorescence staining^20^^,^^21^ (Figure 1I). These results were corroborated through western blot analysis of synovial FLS from patients with OA and RA (Figures 1J and 1K). Collectively, these findings demonstrate that lactate is closely associated with RA pathogenesis and progression, characterized by elevated histone lactylation levels in FLS.

**Figure 1:**
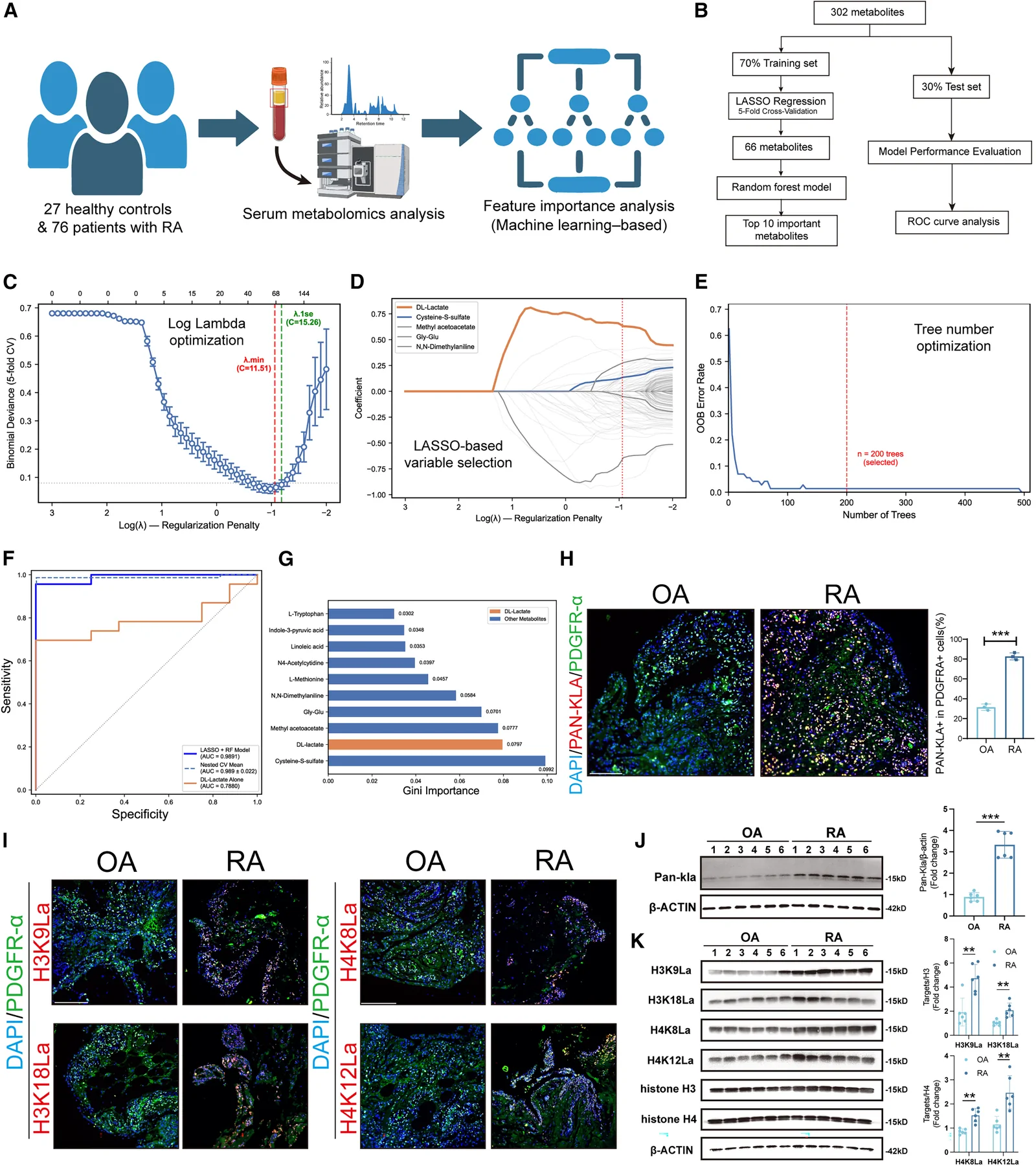
Histone lactylation levels were increased in FLS from RA (A) Schematic diagram of serum metabolomics analysis. Serum samples from 27 healthy controls and 76 patients with rheumatoid arthritis (RA) were collected for serum metabolomics profiling. Machine learning-based variable importance analysis was then performed to identify key metabolites associated with disease diagnosis. (B) The flowchart illustrates the specific methodology for variable screening. A total of 302 metabolites were reduced to 66 through LASSO regression within the training set (70%), and these 66 metabolites were subsequently used to construct a random forest model for the screening of important variables. (C) 5-fold cross-validation plot of the LASSO regression model. Binomial deviance reflects the degree of difference between the model’s predicted outcomes and the actual observed values. Log lambda is the logarithmic transformation of the regularization parameter lambda, used to represent the regularization strength of the model. (D) Coefficients plot of the LASSO regression model. The vertical axis displays the evolution path of feature coefficients with respect to the regularization strength, log lambda. (E) This plot illustrates the trade-off between model complexity (number of trees) and predictive accuracy in a random forest. (F) The ROC curves of the random forest model using LASSO-selected metabolites. Three curves are shown: the multi-metabolite model on the test cohort (AUC = 0.9891; sensitivity: 95.7%, specificity: 87.5%), the nested cross-validation mean (AUC = 0.9889 ± 0.0222), and DL-lactate alone (AUC = 0.7880). (G) The Gini importance of the top ten most discriminant metabolites. DL-lactate ranked second among the 66 LASSO-selected features. (H) Immunofluorescence analysis showed the levels of Pan-Kla in OA and RA synovium (*n* = 3 biological replicates). Scale bars, 100 μm. (I) Immunofluorescence analysis showed the levels of histone H3K9La, H3K18La, H4K8La, and H4K12La in OA and RA synovium (*n* = 3 biological replicates). Statistical analysis was performed using two-tailed unpaired *t* tests. Scale bars, 100 μm. (J) Western blotting analysis of histone Pan-Kla levels in FLS from patients with OA and RA (*n* = 6 biological replicates). Parallel membranes were probed with the indicated antibodies using the same samples. Statistical analysis was performed using two-tailed unpaired *t* tests. (K) Western blotting analysis of histone H3K9La, H3K18La, H4K8La, and H4K12La levels in FLS from patients with OA and RA (*n* = 6 biological replicates). Parallel membranes were probed with the indicated antibodies using the same samples. Statistical analysis was performed using two-tailed unpaired *t* tests. All data are presented as mean ± SD; ∗*p* < 0.05, ∗∗*p* < 0.01, ∗∗∗*p* < 0.001.

### Histone lactylation is elevated in FLS from RA models

To further validate the alterations in histone lactylation in FLS during RA progression, we utilized two widely accepted RA models: the SKG mouse model and the collagen-induced arthritis (CIA) mouse model.^22^^,^^23^ Both animal models exhibit consistent pathological manifestations following disease induction, including ankle joint swelling, synovial hyperplasia, inflammatory cell infiltration, and progressive tissue destruction. These hallmarks closely mirror key features of human RA, particularly in recapitulating the synovial membrane proliferation and osteochondral erosion patterns characteristic of RA. Following the established flowchart, two distinct animal models were constructed (Figure 2A). To investigate the driving role of FLS in RA while minimizing the interference from inflammatory cells during the later stages of the disease, ankle joints were harvested during the early-to-mid inflammatory phases for subsequent extraction of FLS and tissue sections. The results of western blot analysis demonstrated increased histone Pan-Kla levels in FLS from synovial tissues of SKG and CIA mice compared to non-induced controls (Figures 2B and 2C). Furthermore, immunofluorescence staining confirmed the elevated expression of Pan-Kla in FLS (Figures 2D and 2E). Consistent with findings in FLS from patients with RA, the levels of H3K9la, H3K18la, H4K8la, and H4K12la were confirmed to be elevated in FLS from SKG and CIA mice in both western blot analysis and immunofluorescence assays (Figures 2F–2I). Together, these findings highlight a significant upregulation of histone lactylation in FLS during RA, evident in both human patients with RA and induced mouse models.

**Figure 2:**
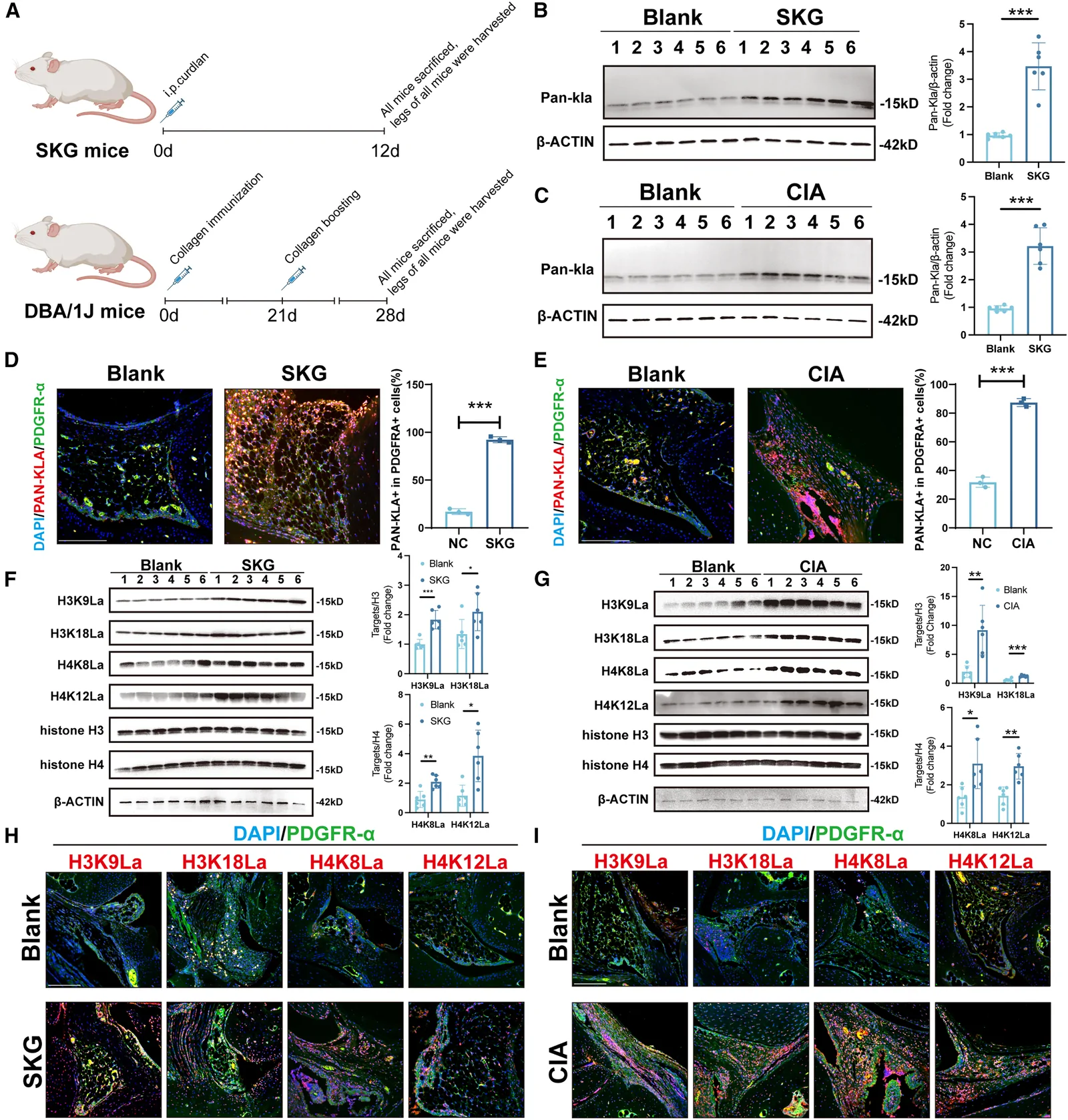
Elevated histone lactylation in FLS from RA models (A) Schematic of SKG and CIA induction. (B) Western blotting analysis of histone Pan-Kla levels in FLS from SKG mice (*n* = 6 biological replicates). Parallel membranes were probed with the indicated antibodies using the same samples. Statistical analysis was performed using two-tailed unpaired *t* tests. (C) Western blotting analysis of histone Pan-Kla levels in FLS from CIA mice (*n* = 6 biological replicates). Parallel membranes were probed with the indicated antibodies using the same samples. Statistical analysis was performed using two-tailed unpaired *t* tests. (D) Immunofluorescence analysis showed the levels of Pan-Kla in SKG mice (*n* = 3 biological replicates). Scale bars, 100 μm. Statistical analysis was performed using two-tailed unpaired *t* tests. (E) Immunofluorescence analysis showed the levels of Pan-Kla in CIA mice (*n* = 3 biological replicates). Scale bars, 100 μm. Statistical analysis was performed using two-tailed unpaired *t* tests. (F) Western blotting analysis of histone H3K9La, H3K18La, H4K8La and H4K12La levels in PDGFR-α+ cells from SKG mice (*n* = 6 biological replicates). Parallel membranes were probed with the indicated antibodies using the same samples. Statistical analysis was performed using two-tailed unpaired *t* tests. (G) Western blotting analysis of histone H3K9La, H3K18La, H4K8La, and H4K12La levels in PDGFR-α+ cells from CIA mice (*n* = 6 biological replicates). Parallel membranes were probed with the indicated antibodies using the same samples. Statistical analysis was performed using two-tailed unpaired *t* tests. (H) Immunofluorescence analysis showed the levels of histone H3K9La, H3K18La, H4K8La, and H4K12La in PDGFR-α+ cells in ankle synovial tissues of SKG mice (*n* = 3 biological replicates). Statistical analysis was performed using two-tailed unpaired *t* tests. Scale bars, 100 μm. (I) Immunofluorescence analysis showed the levels of histone H3K9La, H3K18La, H4K8La, and H4K12La in PDGFR-α+ cells in ankle synovial tissues of CIA mice (*n* = 3 biological replicates). Statistical analysis was performed using two-tailed unpaired *t* tests. Scale bars, 100 μm. All data are presented as mean ± SD; ∗*p* < 0.05, ∗∗*p* < 0.01, ∗∗∗*p* < 0.001.

### Histone lactylation promotes CXCL1 secretion and inflammatory cell recruitment by FLS

SKG mice more closely recapitulate the autoimmune initiation and systemic pathogenesis of RA, making them an optimal model for mechanistic studies. To investigate the functional role of histone lactylation in FLS during RA, FLS were isolated from the ankle synovial tissues of SKG mice and cultured *in vitro*. Sodium lactate was used to stimulate FLS at gradient concentrations (0 mM, 10 mM, 25 mM, and 50 mM) for 24 hours. Western blot analysis revealed a dose-dependent increase in histone Pan-Kla, H3K9la, H3K18la, H4K8la, and H4K12la levels in sodium lactate-treated FLS compared to controls (Figures 3A and S1A). Crucially, sodium lactate itself did not affect FLS viability, and the observed changes in lactylation modification levels could be dissociated from the effects of sodium ion concentration or osmotic pressure (Figures S1C–S1E). To identify the downstream target genes regulated by histone lactylation at the four specific genomic loci, we performed multiplex cleavage under targets and tagmentation sequencing (CUT&Tag-seq) analysis using antibodies targeting the four specific histone lactylation marks: anti-H3K9La, anti-H3K18La, anti-H4K8La and anti-H4K12La. CUT&Tag-seq analysis of control (NC) and sodium lactate-treated FLS (50 mM for 24 hours) showed globally elevated histone lactylation, with peak enrichment at promoter regions (Figures 3B and 3C). Given the established role of promoter histone lactylation in transcriptional activation, we focused on identifying genes associated with differential histone lactylation occupancy across the four specific loci. By comparing the Nalac group with the NC group using CUT&Tag-seq data, we quantified the enrichment levels of H3K9la, H3K18la, H4K8la, and H4K12la at each locus. Genes showing significant differential occupancy (FDR < 0.05, log2 fold change > 1) in the Nalac group were selected as candidate genes, with priority given to those exhibiting concurrent occupancy of multiple histone lactylation marks at their promoter regions. Gene Ontology (GO) enrichment analysis of these upregulated candidates highlighted pathways associated with *monocyte chemotaxis*, *neutrophil chemotaxis*, and *chemokine-mediated signaling pathway* (Figure 3D).

**Figure 3:**
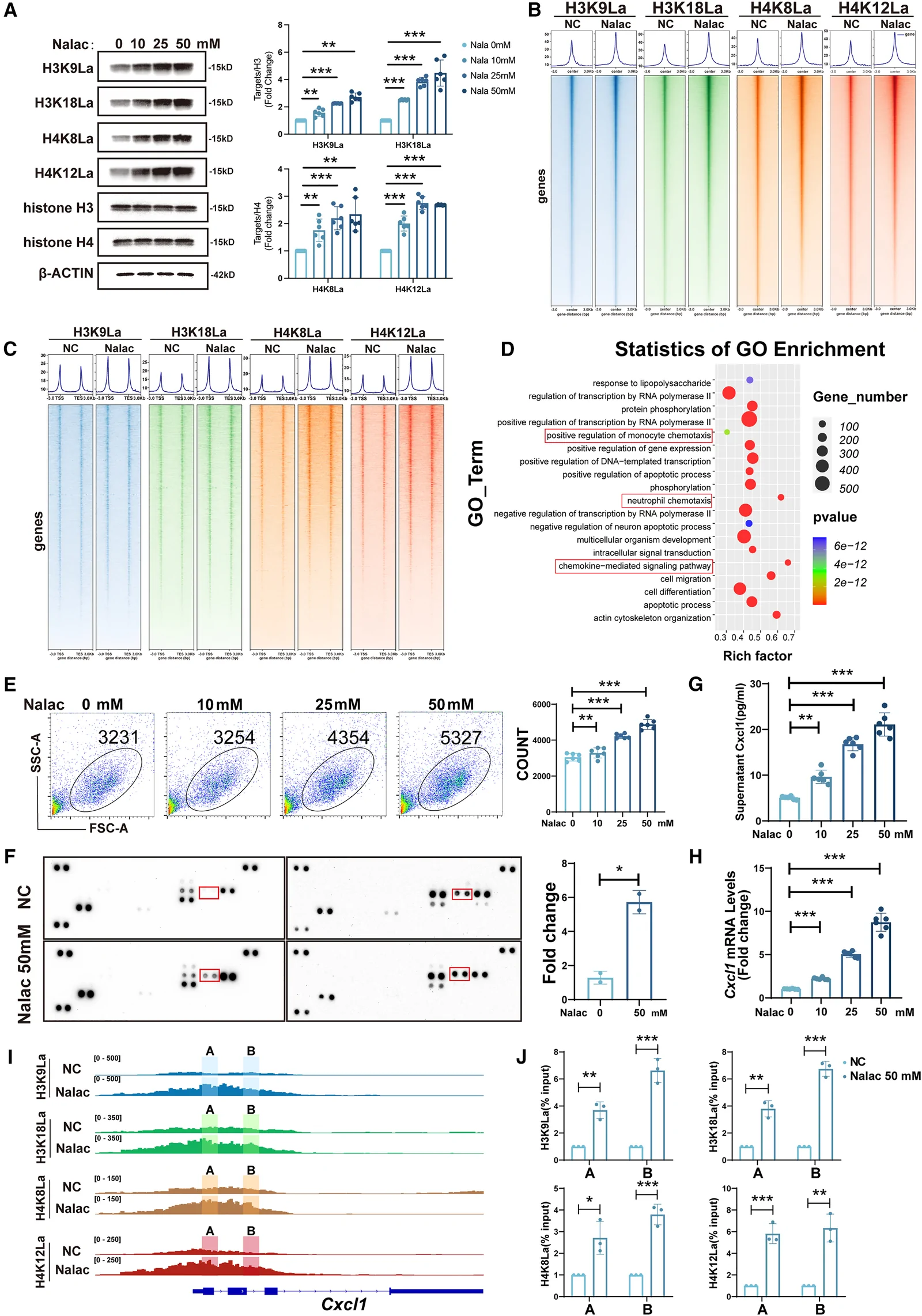
Sodium lactate promotes CXCL1 secretion and the recruitment of inflammatory cells by FLS (A) Western blotting analysis of histone H3K9La, H3K18La, H4K8La, and H4K12La levels in FLS stimulated by different concentrations of sodium lactate (*n* = 6 biological replicates). Parallel membranes were probed with the indicated antibodies using the same samples. Statistical analysis was performed using two-tailed unpaired *t* tests. (B and C) CUT&Tag-seq average binding profiles and heatmaps depicting occupancy of H3K9La, H3K18La, H4K8La, and H4K12La in FLS stimulated by sodium lactate (50 mM). (D) GO biological process analyses increased genes of the CUT&Tag-seq data comparing between the NC and Nalac groups. Bar graph showing the *p* values of the enriched terms. (E) The number of BM cells recruited by FLS in the transwell assay via flow cytometric analysis (*n* = 6 biological replicates). Statistical analysis was performed using two-tailed unpaired *t* tests. (F) Chemokine microarray analysis of chemokines from the supernatant of FLS after sodium lactate stimulation (*n* = 2 biological replicates). Statistical analysis was performed using two-tailed unpaired *t* tests. (G) ELISA analysis showing the protein levels of CXCL1 from the supernatant of FLS after sodium lactate stimulation (*n* = 6 biological replicates). Statistical analysis was performed using two-tailed unpaired *t* tests. (H) RT-qPCR analysis showing the RNA levels of Cxcl1 from FLS after sodium lactate stimulation (*n* = 6 biological replicates). Statistical analysis was performed using two-tailed unpaired *t* tests. (I) Signal traces of CUT&Tag-seq data showing the H3K9La, H3K18La, H4K8La, and H4K12La occupancy on Cxcl1 in the FLS stimulated by sodium lactate. The colored shading shows the different regions. (J) CUT&Tag-qPCR analysis showing the H3K9La, H3K18La, H4K8La, and H4K12La occupancy on Cxcl1 sites A–B in the FLS stimulated by sodium lactate (*n* = 3 biological replicates). Statistical analysis was performed using two-tailed unpaired *t* tests. All data are presented as mean ± SD; ∗*p* < 0.05, ∗∗*p* < 0.01, ∗∗∗*p* < 0.001.

Based on GO analysis, we hypothesized that histone lactylation modifications act as an epigenetic mechanism that modulates the chemotactic capacity of FLS through chemokine signaling pathways. The bone marrow (BM) is a heterogeneous hematopoietic microenvironment containing diverse immune cell populations, including myeloid progenitors (monocytes/macrophages and neutrophils), lymphoid cells, and megakaryocytes. To investigate whether histone lactylation enhances the ability of FLS to recruit immune cells, we co-cultured lactate-pretreated FLS with BM cells in a Transwell system. FLS stimulated with elevated lactate concentrations (≥25 mM) showed a dose-dependent increase in their capacity to attract BM cells, as reflected by the increased number of migrated cells (Figure 3E). Immune cell recruitment is primarily mediated by chemokines, and GO analysis of upregulated genes revealed significant enrichment in chemokine-related pathways, suggesting a molecular basis for their regulatory role. Multiplex chemokine array analysis of culture supernatants showed that lactate preconditioning markedly increased chemokine secretion by FLS (Figure 3F). CXCL1 was identified as the most significantly upregulated chemokine, which was confirmed by ELISA (Figure 3G). To determine whether this upregulation occurred at both the transcriptional and translational levels, we performed reverse transcription quantitative PCR (RT-qPCR) and western blotting, confirming that CXCL1 was upregulated at both the mRNA and protein levels (Figures 3H and S1B). CUT&Tag-seq and CUT&Tag-qPCR analyses further revealed increased H3K9la, H3K18la, H4K8la, and H4K12la occupancy at the *Cxcl1* promoter in Nalac-treated FLS (Figures 3I and 3J). To determine whether CXCL1 directly mediates BM cell recruitment by FLS, we knocked down CXCL1 expression using small interfering RNA (siRNA) or neutralized CXCL1 with a monoclonal antibody. Both interventions significantly reduced BM cell migration compared with controls, and this reduction was restored by CXCL1 supplementation, as quantified by Transwell assays (Figures S2A and S2B). This functional validation establishes that CXCL1 is a critical effector molecule downstream of FLS-derived chemokine signaling. Collectively, these results demonstrate that histone lactylation in FLS epigenetically enhances CXCL1 expression, thereby driving immune cell recruitment through a chemokine-dependent mechanism.

### CXCL1 expression and secretion are elevated in FLS during RA

To examine the relationship between CXCL1 expression and RA, publicly available datasets from the Gene Expression Omnibus (GEO) were analyzed to compare synovial *CXCL1* mRNA levels in patients with OA and RA, as well as in RA models. Analysis of GSE36700 revealed significantly higher *CXCL1* mRNA expression in the knee joint synovium of patients with RA than in that of patients with OA^24^ (Figure 4A). Similarly, Cxcl1 mRNA was notably upregulated in the synovium of human tumor necrosis factor transgenic (huTNFtg) mice and adjuvant-induced arthritis (AIA) rats (GSE210887 and GSE276556)^25^^,^^26^ (Figure 4B). To further validate these findings in the inflamed ankle synovium of SKG and CIA models, we quantified CXCL1 at both the protein and mRNA levels using ELISA and RT-qPCR, respectively. Both CXCL1 protein and mRNA were significantly upregulated in the synovium of these models (Figures 4C, S3A and S3B). Immunohistochemistry (IHC) further confirmed that CXCL1 was elevated in synovial tissues from patients with RA, SKG mice, and CIA mice (Figures 4D–4F). Although our *in vitro* and *in vivo* experiments suggest that FLS secrete CXCL1 under disease conditions, direct evidence identifying FLS as the predominant source of CXCL1 within the complex *in vivo* synovial microenvironment has been limited. Therefore, we leveraged a single-cell RNA sequencing dataset generated by Fan Zhang et al. that covers synovial tissue from 36 patients with RA and 15 patients with OA.^27^ Analysis of this dataset identified four major cell types—B cells, T cells, monocytes, and FLS—with their lineage-defining marker genes shown (Figures 4G and 4H). Examination of CXCL1 expression revealed only a subtle difference in average expression levels between RA and OA samples (Figure 4I). However, aggregate expression analysis, which accounts for cell abundance, showed a significant increase in total CXCL1 transcripts in RA synovium compared with OA (Figure 4I). Furthermore, interrogation of CXCL1 expression across synovial cell subpopulations identified FLS as the cell type with the highest CXCL1 expression in RA synovium (Figure 4J). Together, these findings provide evidence that FLS are a major source of CXCL1 in the RA joint *in vivo*.

**Figure 4:**
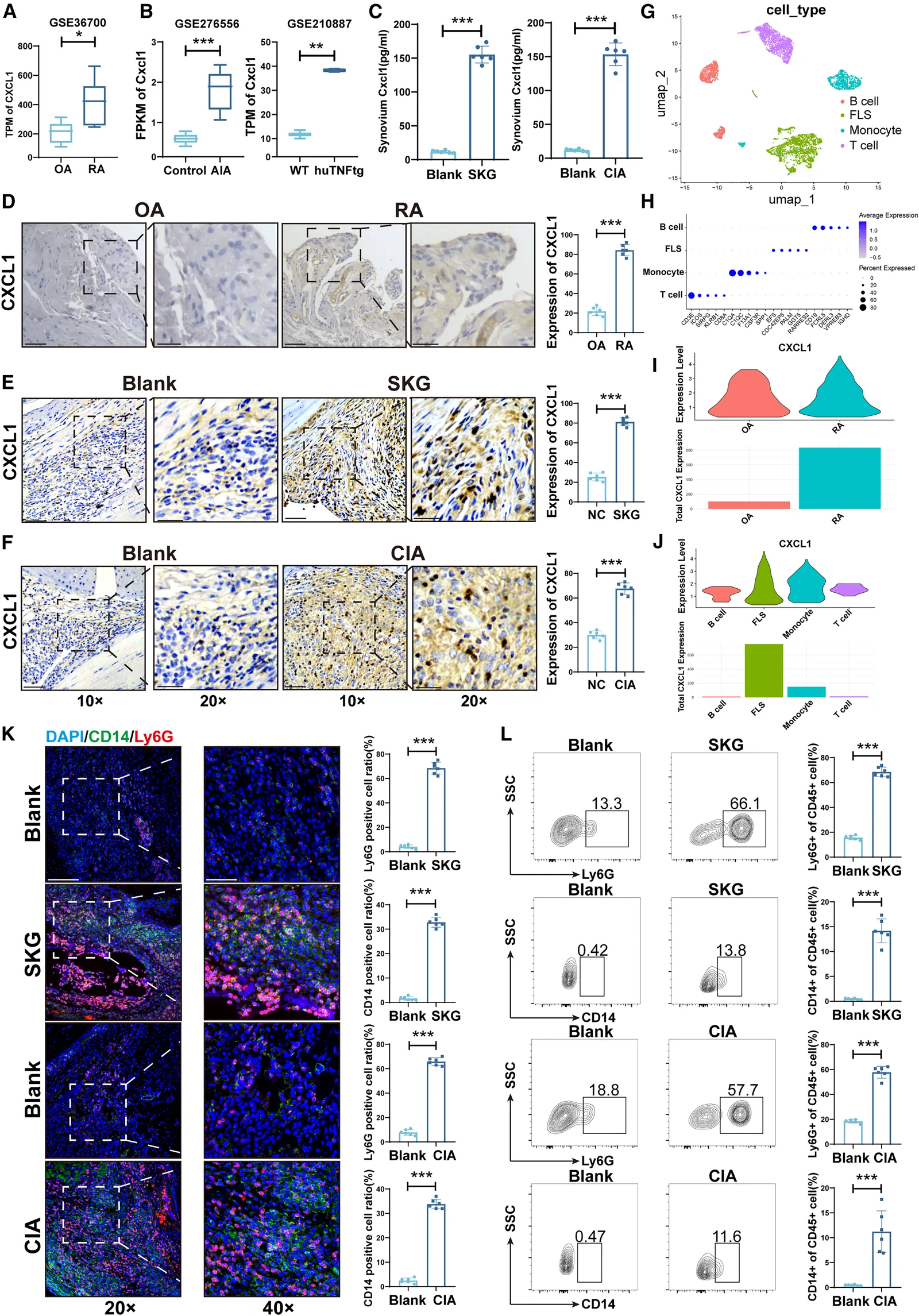
FLS recruited inflammatory cells via Cxcl1 in RA (A) Expression of CXCL1 in synovial tissues of patients with OA and RA from a public dataset (GSE36700). Statistical analysis was performed using two-tailed unpaired *t* tests. (B) Expression of Cxcl1 in synovial tissues of huTNFtg and AIA models compared to NC groups from public datasets (GSE210887 and GSE276556). Statistical analysis was performed using two-tailed unpaired *t* tests. (C) ELISA analysis showing the protein levels of CXCL1 in synovial tissue from SKG and CIA mice (*n* = 6 biological replicates). Statistical analysis was performed using two-tailed unpaired *t* tests. (D) Immunohistochemistry analysis showing the protein levels of CXCL1 in synovial tissue from OA and RA (*n* = 6 biological replicates). 10 × scale bars, 400 μm; 20 × scale bars, 200 μm. Statistical analysis was performed using two-tailed unpaired *t* tests. (E and F) Immunohistochemistry analysis showing the protein levels of CXCL1 in synovial tissue from SKG and CIA mice (*n* = 6 biological replicates). 10 × scale bars, 400 μm; 20 × scale bars, 200 μm. Statistical analysis was performed using two-tailed unpaired *t* tests. (G) Dimplot clustering diagram of synovial tissue samples from patients with RA and OA based on single-cell RNA sequencing. (H) Top five marker genes by expression level in T cells, B cells, monocytes and FLS populations. (I) The violin plot displays the average cell expression levels of CXCL1 RNA in patients with OA and RA; the bar chart shows the overall cell expression levels of CXCL1 RNA in patients with OA and RA. (J) The violin plot displays the average cell expression levels of CXCL1 RNA in T cells, B cells, monocytes, and FLS populations; the bar chart shows the overall cell expression levels of CXCL1 RNA in T cells, B cells, monocytes, and FLS populations. (K) Immunofluorescence showing Ly6G+ and CD14^+^ cells in the synovial tissues of SKG and CIA mice and Blank groups (*n* = 6 biological replicates). 20 × scale bars, 200 μm; 40 × scale bars, 100 μm. Statistical analysis was performed using two-tailed unpaired *t* tests. (L) Flow cytometry assay showing Ly6G+ and CD14^+^ cell ratios in the synovial tissue of SKG and CIA mice and blank groups (*n* = 6 biological replicates). Statistical analysis was performed using two-tailed unpaired *t* tests. All data are presented as mean ± SD; ∗*p* < 0.05, ∗∗*p* < 0.01, ∗∗∗*p* < 0.001.

Since CXCL1 predominantly recruits neutrophils and monocytes, we performed immunofluorescence to assess these populations in the ankle joint synovium of SKG and CIA mice.^28^ Neutrophils and monocytes were identified using Ly6G and CD14 as surface markers, respectively. Immunofluorescence staining revealed a marked increase in the proportion of Ly6G^+^ neutrophils and CD14^+^ monocytes in the inflamed synovium of SKG and CIA mice compared with non-arthritic controls (Figure 4K). To further validate these findings, we quantified neutrophils and monocytes by flow cytometry using the same markers. Consistent with the immunofluorescence results, flow cytometry also showed a significant increase in these populations in the synovium of SKG and CIA mice relative to controls (Figure 4L). Together, these findings demonstrate a significant upregulation of CXCL1 expression and secretion in the RA synovium, which facilitates the recruitment of neutrophils and monocytes, and underscores its critical role in orchestrating the inflammatory microenvironment.

### Prophylactic CXCL1 neutralization prevents joint inflammation and bone destruction in SKG and CIA mice

To evaluate the prophylactic therapeutic potential of CXCL1 blockade in RA, we administered a CXCL1-neutralizing antibody via tail vein injection in SKG and CIA mice. SKG mice received the antibody one day before arthritis induction and again on day 12 post-induction, whereas CIA mice received the same antibody one day before collagen immunization and before collagen boost—corresponding to their two-phase immune sensitization protocol (Figure 5A). Immunofluorescence showed fewer neutrophils and monocytes in the synovium of antibody-treated mice than in IgG controls (Figure 5B). This reduction was confirmed by flow cytometry, which revealed markedly lower frequencies of neutrophils (Ly6G^+^) and monocytes (CD14^+^) within the inflamed synovial tissue (Figures S4A and S4B). In RA, CXCL1-recruited neutrophils are critical mediators of synovial inflammation and tissue injury,^29^ whereas CXCL1-recruited monocytes promote osteoclastogenesis and exacerbate bone erosion by differentiating into osteoclasts.^30^ To evaluate the impact of CXCL1 neutralization on disease progression, we assessed joint inflammation and bone destruction. CXCL1-neutralized SKG and CIA mice had significantly lower arthritis scores and reduced hind paw thickness than controls (Figures 5C and 5D). Histological assessment—including macroscopic imaging, H&E staining and safranin O-fast green staining—showed reduced synovial inflammation and cartilage erosion in CXCL1-neutralized mice (Figure 5E). Furthermore, micro-computed tomography (CT) imaging and tartrate-resistant acid phosphatase (TRAP) staining revealed fewer osteoclasts and less bone destruction in the ankle joints of treated mice (Figure 5F). Together, these results indicate that CXCL1 neutralization attenuates both neutrophil-driven synovial inflammation and osteoclast-mediated bone destruction, supporting its potential as a prophylactic therapeutic strategy for RA.

**Figure 5:**
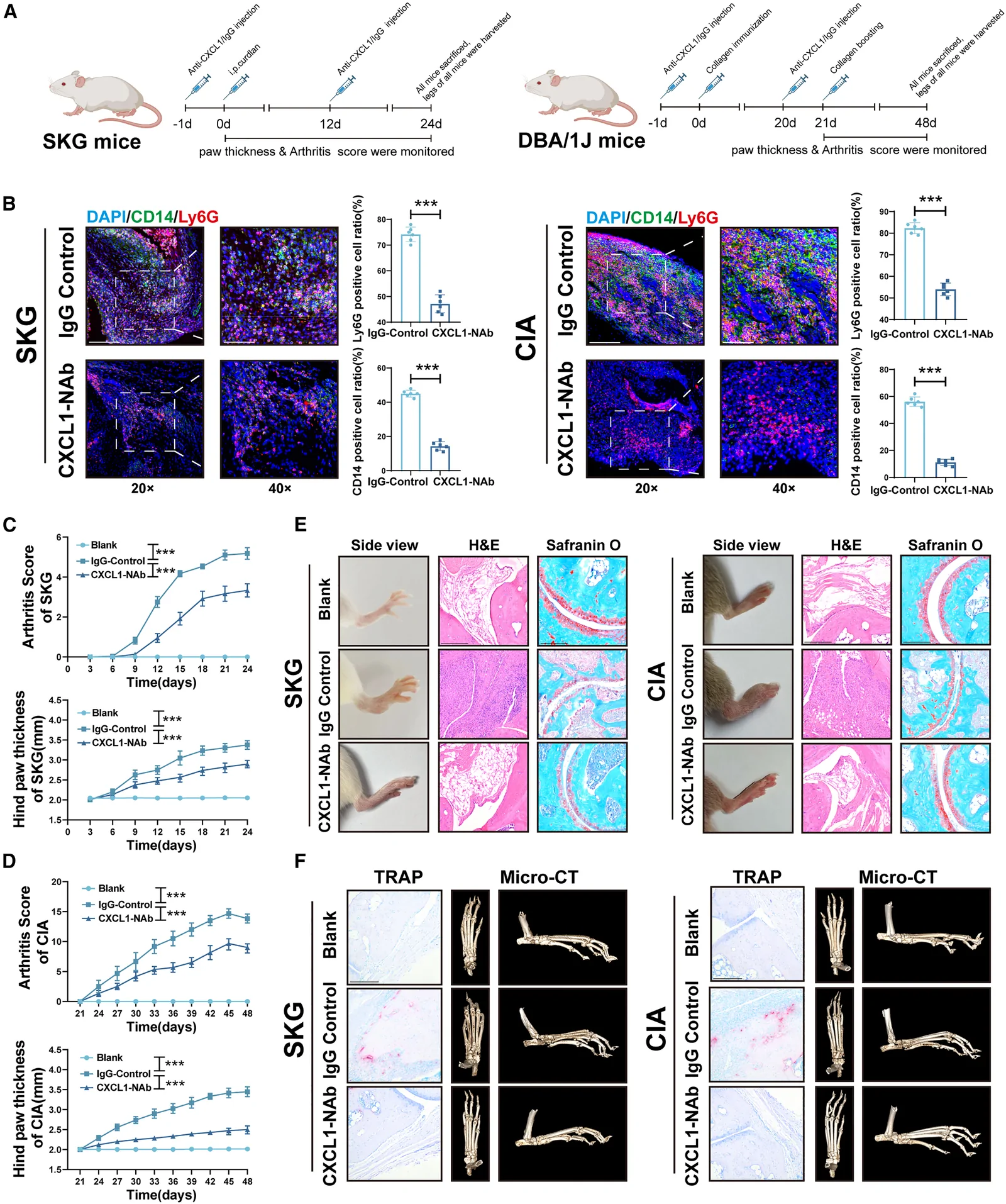
CXCL1-neutralizing antibody treatment attenuated joint inflammation and bone destruction in SKG and CIA mice (A) Schematic of SKG and CIA induction and CXCL1 neutralizing antibody (CXCL1 NAb) administration. IgG from rabbit was used as control. (B) Immunofluorescence assay showing Ly6G+ and CD14^+^ cells in the synovial tissue of SKG and CIA mice treated with CXCL1 NAb or IgG (*n* = 6 biological replicates). 20 × scale bars, 200 μm; 40 × scale bars, 100 μm. Statistical analysis was performed using two-tailed unpaired *t* tests. (C) The arthritis score and paw thickness of the CXCL1 NAb groups and IgG groups in SKG mice (*n* = 6 biological replicates). The *p* value shown in the source data was based on a two-way ANOVA followed by Bonferroni’s post hoc comparisons. (D) The arthritis score and paw thickness of the CXCL1 NAb groups and IgG groups in CIA mice (*n* = 6 biological replicates). The *p* value shown in the source data was based on a two-way ANOVA followed by Bonferroni’s post hoc comparisons. (E) Macroscopic images, H&E staining and safranin O staining of the ankle joints from SKG and CIA mice treated with IgG or CXCL1 NAb (*n* = 3 biological replicates). Scale bars, 400 μm. (F) TRAP staining and micro-CT images of the ankle joints from SKG and CIA mice treated with IgG or CXCL1 NAb (*n* = 3 biological replicates). Scale bars, 400 μm. All data are presented as mean ± SD; ∗*p* < 0.05, ∗∗*p* < 0.01, ∗∗∗*p* < 0.001.

### Prophylactic LDHA inhibition reduces joint inflammation and bone destruction in SKG and CIA mice

Lactate dehydrogenase A (LDHA), a central rate-limiting enzyme in glycolysis, catalyzes the conversion of pyruvate to lactate and plays a key role in metabolic reprogramming during inflammation.^31^ Pharmacological inhibition of LDHA with selective inhibitors, such as GNE140, effectively reduces lactate production *in vitro* and *in vivo*.^32^ GNE140, a highly specific small-molecule LDHA inhibitor that targets LDHA-NADH complex, has been widely used in preclinical studies to suppress lactate production.^33^ To investigate whether LDHA inhibition ameliorates RA, we administered GNE140 by oral gavage to SKG and CIA mice (Figures S5A and S5B). GNE140 treatment significantly reduced lactate levels in the synovium of ankle joints compared to vehicle controls (Figures S5C and S5D). Immunofluorescence revealed reduced H3K9la, H3K18la, H4K8la, and H4K12la in the synovium of GNE140-treated mice (Figures 6A and 6B), suggesting that histone lactylation decreases in parallel with local lactate levels. IHC and ELISA showed that GNE140 significantly reduced CXCL1 levels: IHC showed decreased CXCL1 in the inflamed synovium, and ELISA quantification confirmed lower CXCL1 in synovial fluid compared with vehicle-treated controls (Figures 6C and 6D). Furthermore, immunofluorescence and flow cytometry revealed that GNE140 markedly reduced neutrophil (Ly6G^+^) and monocyte (CD14^+^) infiltration in the synovium of SKG and CIA mice compared with vehicle controls (Figures 6E, 6F, S5E, and S5F). Thus, GNE140 suppresses lactylation-dependent CXCL1 secretion and inhibits neutrophil and monocyte infiltration into the synovium. Clinically, GNE140 treatment reduced arthritis scores and hind paw thickness in SKG and CIA mice (Figures 6G and 6H). Macroscopic imaging, H&E staining, and safranin O-fast green staining confirmed reduced arthritis severity (Figures 6I and 6J). Micro-CT and TRAP staining revealed less bone destruction and fewer osteoclasts in the joints of GNE140-treated SKG and CIA mice (Figures 6K and 6L). Together, these findings show that LDHA inhibition reduces synovial lactate accumulation, decreases histone lactylation in FLS and CXCL1 secretion, and alleviates joint inflammation and osteoclast-mediated bone destruction.

**Figure 6:**
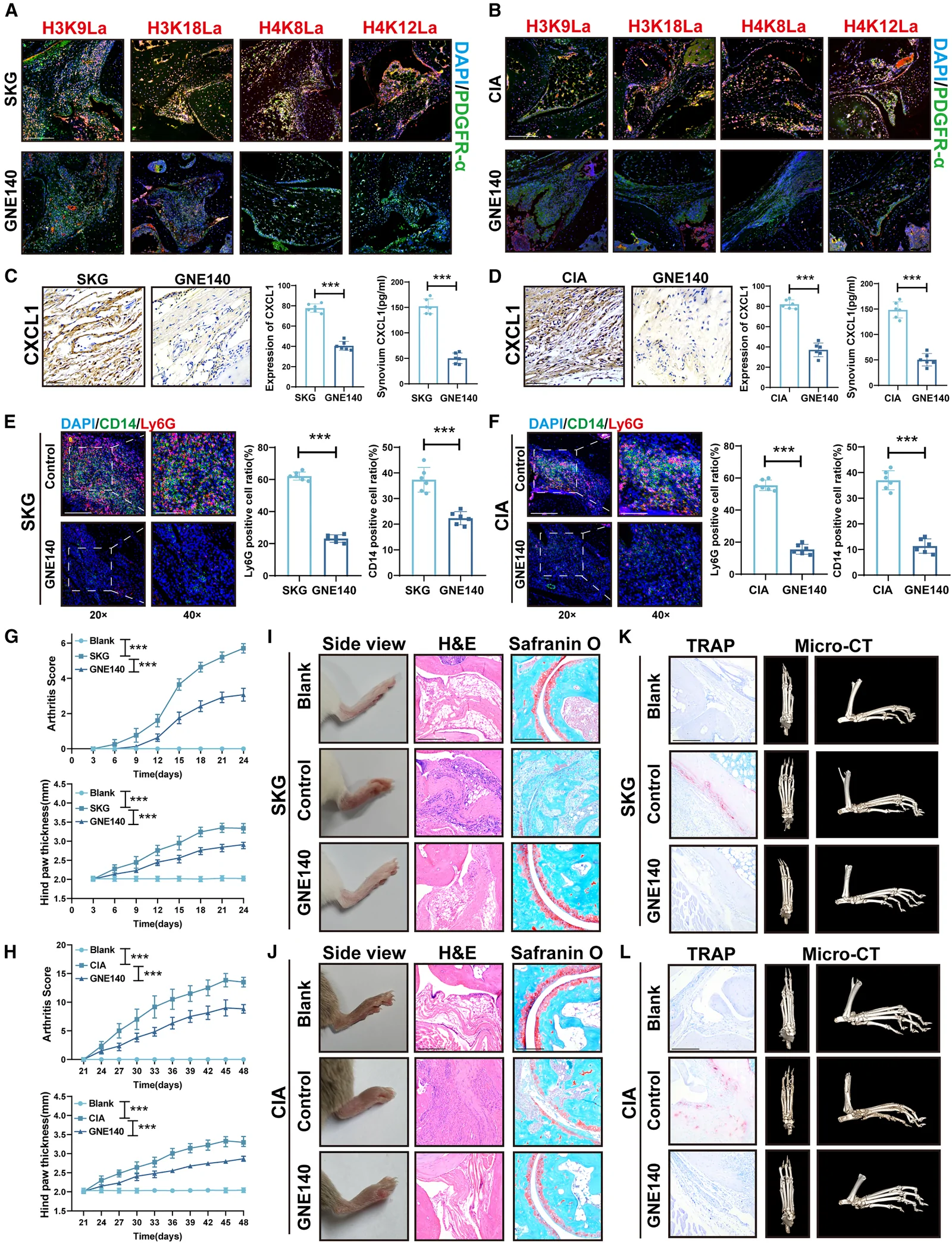
GNE140 treatment attenuated joint inflammation and bone destruction in SKG and CIA mice (A) Immunofluorescence showing the levels of histone H3K9La, H3K18La, H4K8La, and H4K12La in PDGFR-α+ cells in ankle synovial tissues of SKG mice treated with GNE140 or vehicle (*n* = 3 biological replicates). Scale bars, 200 μm. (B) Immunofluorescence showing the levels of histone H3K9La, H3K18La, H4K8La, and H4K12La in PDGFR-α+ cells in ankle synovial tissues of CIA mice treated with GNE140 or vehicle (*n* = 3 biological replicates). Scale bars, 200 μm. (C) Immunohistochemistry and ELISA showing the CXCL1 levels in ankle synovial tissues and fluid of SKG mice treated with GNE140 or vehicle (*n* = 6 biological replicates). Scale bars, 400 μm. Statistical analysis was performed using two-tailed unpaired *t* tests. (D) Immunohistochemistry and ELISA showing the CXCL1 levels in ankle synovial tissues and fluid of CIA mice treated with GNE140 or vehicle (*n* = 6 biological replicates). Scale bars, 400 μm. Statistical analysis was performed using two-tailed unpaired *t* tests. (E) Immunofluorescence showing Ly6G+ and CD14^+^ cells in the synovial tissues of SKG mice treated with GNE140 or vehicle (*n* = 6 biological replicates). 20 × scale bars, 200 μm; 40 × scale bars, 100 μm. Statistical analysis was performed using two-tailed unpaired *t* tests. (F) Immunofluorescence showing Ly6G+ and CD14^+^ cells in the synovial tissues of CIA mice treated with GNE140 or vehicle (*n* = 6 biological replicates). 20 × scale bars, 200 μm; 40 × scale bars, 100 μm. Statistical analysis was performed using two-tailed unpaired *t* tests. (G) The arthritis score and paw thickness of the GNE140 groups and vehicle groups in SKG mice (*n* = 6 biological replicates). The *p* value shown in the source data was based on a two-way ANOVA followed by Bonferroni’s post hoc comparisons. (H) The arthritis score and paw thickness of the GNE140 groups and vehicle groups in CIA mice (*n* = 6 biological replicates). The *p* value shown in the source data was based on a two-way ANOVA followed by Bonferroni’s post hoc comparisons. (I) Macroscopic images, H&E staining and safranin O staining of the ankle joints from SKG mice treated with GNE140 or vehicle (*n* = 3 biological replicates). Scale bars, 400 μm. (J) Macroscopic images, H&E staining, and safranin O staining of the ankle joints from CIA mice treated with GNE140 or vehicle (*n* = 3 biological replicates). Scale bars, 400 μm. (K) TRAP staining and micro-CT images of the ankle joints from SKG mice treated with GNE140 or vehicle (*n* = 3 biological replicates). (L) TRAP staining and micro-CT images of the ankle joints from CIA mice treated with GNE140 or vehicle (*n* = 3 biological replicates). Scale bars, 400 μm. See also Figure S5. All data are presented as mean ± SD; ∗*p* < 0.05, ∗∗*p* < 0.01, ∗∗∗*p* < 0.001.

### FDA-approved drug stiripentol attenuates joint inflammation and bone destruction in SKG and CIA mice

Although no LDHA inhibitors have been approved for clinical use, the FDA-approved antiepileptic drug stiripentol has recently been identified as a repurposable agent with LDHA-inhibitory activity. Originally approved for refractory epilepsy, stiripentol suppresses lactate production by directly modulating LDHA enzymatic function, supporting further investigation of its application in metabolic-inflammatory disorders.^34^ To investigate whether stiripentol alleviates RA by reducing lactate production, SKG and CIA mice received stiripentol by oral gavage (Figures S6A and S6B). Stiripentol did not significantly affect body weight or liver and kidney histology (Figures S6C–S6F). Lactate levels in the synovium of ankle joints were significantly reduced in stiripentol-treated SKG and CIA mice (Figures S6G and S6H). Immunofluorescence revealed reduced H3K9la, H3K18la, H4K8la and H4K12la in the FLS of ankle joints from stiripentol-treated mice compared with vehicle controls (Figures 7A and 7B). Immunohistochemistry and ELISA showed a significantly lower CXCL1 expression in the synovium and synovial fluid of stiripentol-treated SKG and CIA mice (Figures 7C and 7D). Immunofluorescence and flow cytometry analyses demonstrated reduced infiltration in the ankle synovium (Figures 7E, 7F, S6I, and S6J). Furthermore, stiripentol significantly reduced arthritis scores and hind paw thickness in SKG and CIA mice (Figures 7G and 7H). Macroscopic imaging, H&E staining, and safranin O-fast green staining confirmed reduced arthritis severity in stiripentol-treated SKG and CIA mice (Figure S7A). Micro-CT and TRAP staining showed less bone destruction and fewer osteoclasts in the joints of these mice (Figure S7B). Together, these results highlight the therapeutic potential of stiripentol, an FDA-approved antiepileptic drug, as a repurposable LDHA inhibitor for RA. Stiripentol effectively reduced histone lactylation, suppressed CXCL1 secretion, attenuated immune cell recruitment, and alleviated joint inflammation and bone destruction.

**Figure 7:**
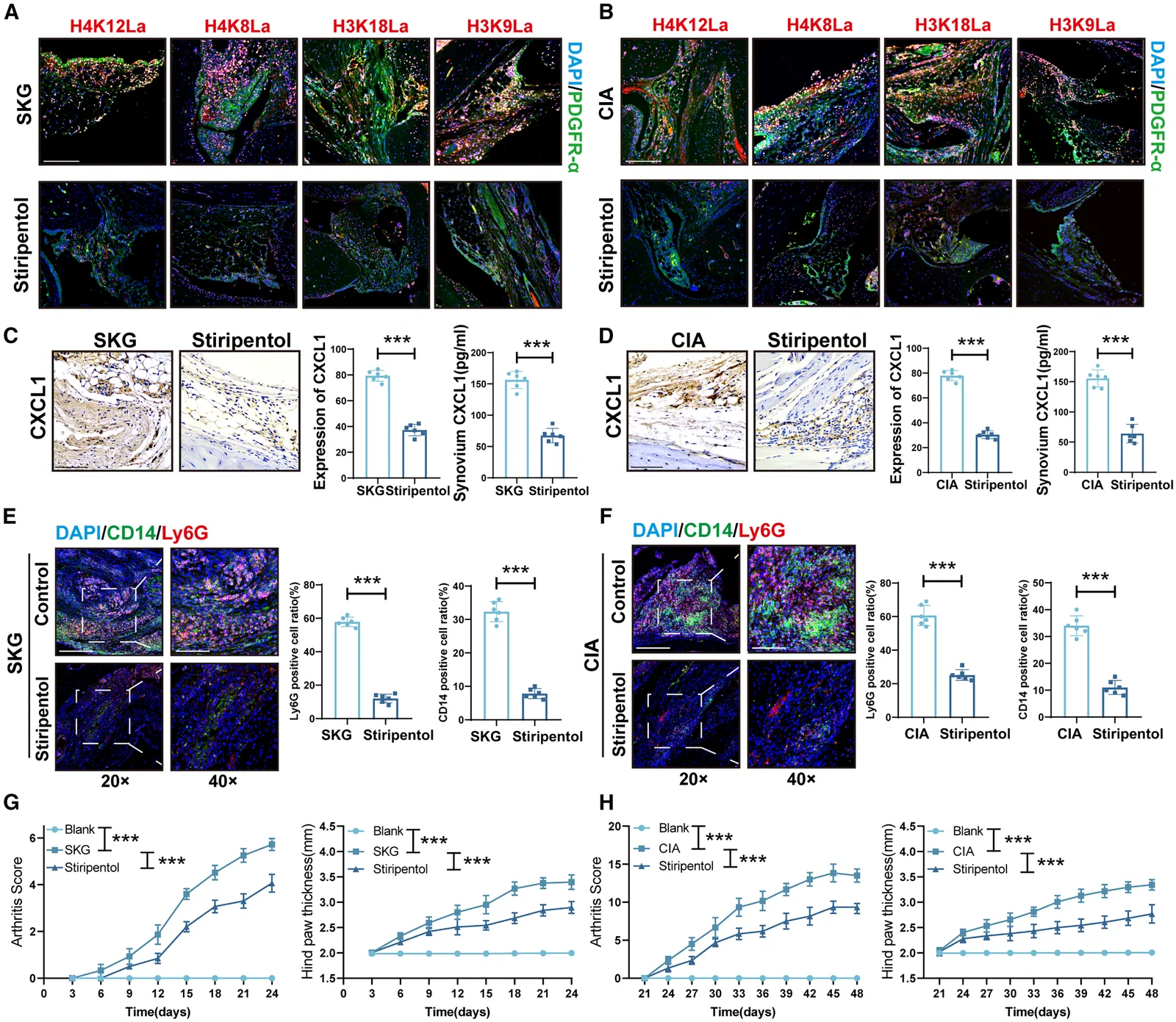
Stiripentol treatment attenuated joint inflammation and bone destruction in SKG and CIA mice (A) Immunofluorescence showing the levels of histone H3K9La, H3K18La, H4K8La, and H4K12La in PDGFR-α+ cells in ankle synovial tissues of SKG mice treated with stiripentol or vehicle (*n* = 3 biological replicates). Scale bars, 200 μm. (B) Immunofluorescence showing the levels of histone H3K9La, H3K18La, H4K8La, and H4K12La in PDGFR-α+ cells in ankle synovial tissues of CIA mice treated with stiripentol or vehicle (*n* = 3 biological replicates). Scale bars, 200 μm. (C) Immunohistochemistry and ELISA showing the CXCL1 levels in ankle synovial tissues and fluid of SKG mice treated with stiripentol or vehicle (*n* = 6 biological replicates). Scale bars, 400 μm. Statistical analysis was performed using two-tailed unpaired *t* tests. (D) Immunohistochemistry and ELISA showing the CXCL1 levels in ankle synovial tissues and fluid of CIA mice treated with stiripentol or vehicle (*n* = 6 biological replicates). Scale bars, 400 μm. Statistical analysis was performed using two-tailed unpaired *t* tests. (E) Immunofluorescence showing Ly6G+ and CD14^+^ cells in the synovial tissues of SKG mice treated with stiripentol or vehicle (*n* = 6 biological replicates). 20 × scale bars, 200 μm; 40 × scale bars, 100 μm. Statistical analysis was performed using two-tailed unpaired *t* tests. (F) Immunofluorescence showing Ly6G+ and CD14^+^ cells in the synovial tissues of CIA mice treated with stiripentol or vehicle (*n* = 6 biological replicates). 20 × scale bars, 200 μm; 40 × scale bars, 100 μm. Statistical analysis was performed using two-tailed unpaired *t* tests. (G) The arthritis score and paw thickness of the stiripentol groups and vehicle groups in SKG mice (*n* = 6 biological replicates). The *p* value shown in the source data was based on a two-way ANOVA followed by Bonferroni’s post hoc comparisons. (H) The arthritis score and paw thickness of the stiripentol groups and vehicle groups in CIA mice (*n* = 6 biological replicates). The *p* value shown in the source data was based on a two-way ANOVA followed by Bonferroni’s post hoc comparisons. See also Figure S6. All data are presented as mean ± SD; ∗*p* < 0.05, ∗∗*p* < 0.01, ∗∗∗*p* < 0.001.

## Discussion

Metabolic reprogramming toward glycolytic dominance and lactate accumulation is a hallmark of RA.^13^ Elevated glycolysis and subsequent lactate accumulation have been implicated in RA progression.^13^^,^^35^ Serum lactate levels are elevated in patients with RA, particularly in early disease.^36^ In line with these observations, we developed a clinical prediction model combining LASSO regression and random forest algorithms and demonstrated the diagnostic potential of serum lactate for RA. Together, these findings support a role for lactate in RA pathogenesis, particularly at disease onset. However, the precise mechanisms by which lactate mediates RA pathogenesis remain unclear and require further investigation.

Lactylation, a post-translational modification first described in 2019, represents a significant biological advance.^14^ This modification affects a broad range of proteins, including histones, signaling molecules and enzymes, and exerts diverse regulatory functions in both physiological and pathological processes—potentially accounting for many of the mechanisms by which lactate mediates its effects.^37^ However, its role in RA remains largely unexplored. Here, we show that histone lactylation is markedly elevated in synovial FLS within RA lesions, suggesting that histone lactylation may contribute to RA pathogenesis and warrants further investigation.

FLS in RA are not merely passive responders to the inflammatory milieu; rather, they acquire an activated and aggressive phenotype that actively shapes the lesion microenvironment.^7^ A hallmark of pathogenic FLS is their ability to recruit leukocytes, contributing to the establishment and maintenance of a pro-inflammatory microenvironment through chemokine secretion.^38^ Lactate-activated fibroblasts were shown to secrete IL-8, which recruited tumor-associated macrophages (TAMs) and reshaped the tumor microenvironment, promoting lung cancer progression.^39^ Here we found that histone lactylation enhances the chemotactic capacity of FLS by upregulating CXCL1 transcription. Consistent with our findings, elevated CXCL1 levels have been reported in patients with RA and in mouse models of the disease.^24^^,^^25^^,^^26^ Notably, plasma CXCL1 levels in patients with RA correlate positively with the disease activity score in 28 joints (DAS28), a key clinical indicator of disease severity.^40^ CXCL1 primarily activates CXCR2 to recruit neutrophils and monocytes, both essential components of the innate immune system.^28^ Neutrophils are rapidly recruited to inflamed joints, where they release pro-inflammatory mediators that exacerbate tissue damage and attract additional immune cells.^41^ They also form neutrophil extracellular traps (NETs), which enhance T cell activation and sustain chronic inflammation.^42^ Furthermore, neutrophils modulate Th17 cell differentiation, linking innate and adaptive immune responses in RA.^43^ Once in the inflamed joint, monocytes differentiate into macrophages that secrete TNF-α and IL-6, sustaining inflammation and driving joint damage.^44^ Some monocytes also differentiate into osteoclasts, contributing to bone erosion by degrading bone matrix.^45^^,^^46^ As antigen-presenting cells (APCs), monocytes play a key role in activating T cells, thereby linking innate and adaptive immune dysfunction in RA.^47^ Thus, neutrophils and monocytes not only mediate the initial innate immune response but also regulate adaptive immunity.^48^ Given its role in immune cell recruitment, CXCL1 is a promising therapeutic target for RA. In two established mouse models of RA, CXCL1 neutralization effectively inhibited neutrophil and monocyte recruitment and infiltration, leading to significantly reduced inflammation and bone destruction.

The hyperactive glycolytic metabolism within the disease microenvironment leads to excessive lactate accumulation, which drives widespread lactylation.^14^ We identified a significant upregulation of histone lactylation marks, particularly at the Cxcl1 promoter. These observations suggest that targeting a single lactylation enzyme or modification may be insufficient owing to potential compensatory effects from other isoenzymes and downstream lactylation events. To address this, we focused on suppressing lactate production to inhibit pan-lactylation in FLS and reduce CXCL1 levels, offering a potential therapeutic strategy for RA. In two RA models, a prophylactic regimen was used in which LDHA inhibitors were administered at the time of arthritis induction. Early intervention suppressed FLS metabolic reprogramming and epigenetic remodeling. LDHA inhibition reduced lactate levels in arthritic lesions, histone lactylation and CXCL1 secretion. Consequently, this weakened the role of FLS as a signaling hub for immune cell recruitment, leading to reduced immune cell infiltration, attenuated joint inflammation and decreased bone destruction. Critically, targeting the chemotactic function of FLS reduced neutrophil and monocyte infiltration without causing systemic immunosuppression, thereby avoiding the adverse effects of conventional therapies. Together, these findings support the translational potential of this therapeutic approach.

In conclusion, this proof-of-concept study demonstrates that targeting histone lactylation in FLS suppresses their recruitment phenotype in RA, reducing CXCL1 secretion and decreasing the infiltration of key innate immune cells. This approach attenuates arthritis severity and joint destruction, supporting a promising therapeutic strategy for RA. Although CXCL1-neutralizing antibodies and selective LDH inhibitors such as GNE140 have not yet been approved for clinical use, we evaluated stiripentol, an FDA-approved antiepileptic drug with LDH-inhibitory activity, as a therapeutic strategy for targeting LDH.^33^^,^^34^ Previous studies have shown that stiripentol inhibits abnormal angiogenesis by suppressing LDH activity, supporting its potential for treating RA-related conditions.^49^ Here, we show that stiripentol effectively reduced lactate production, suppressed histone lactylation-mediated CXCL1 secretion, and decreased immune cell recruitment and infiltration in two RA models, leading to alleviation of arthritis severity and bone destruction. These findings highlight the potential of repurposing stiripentol, an FDA-approved drug with established clinical safety and LDHA-inhibitory activity, for the treatment of RA. Further clinical investigation into stiripentol’s therapeutic potential in RA is essential for future research.

### Limitations of the study

Several limitations of this study should be noted. First, the CXCL1 neutralizing antibody was administered prophylactically; whether CXCL1 blockade can reverse established disease remains to be determined. Furthermore, blockade of the downstream receptor CXCR2 has not been sufficiently validated. Second, no full dose-response titration or formal neutralization efficiency assessment was performed; antibody doses were selected based on published chemokine neutralization protocols in murine models. Third, both GNE140 and stiripentol were administered systemically and may directly affect the activity of multiple cell subsets, including neutrophils, T cells, macrophages, chondrocytes, and osteocytes. Future studies utilizing FLS-specific Ldha knockout or adoptive transfer with conditioned media from drug-treated FLS are needed to definitively establish whether intrinsic lactate metabolism within FLS is the dominant driver of immune cell recruitment in arthritis.

## Resource availability

### Lead contact

Requests for further information and resources should be directed to and will be fulfilled by the lead contact, Guiwen Ye (yegw@mail.sysu.edu.cn).

### Materials availability

This study did not generate new unique reagents.

### Data and code availability

• The CUT&Tag-seq data have been deposited at GEO: GSE327688. The RNA-sequencing and microarray data analyzed in this study are available from the GEO under the accession numbers: GSE36700, GSE210887, and GSE276556. The metabolomics data analyzed in this study are available from MetaboLights under the accession number MTBLS5297.
• This paper does not report original code.
• Any additional information required to reanalyze the data reported in this paper is available from the lead contact upon request.

## Acknowledgments

This study was supported by the Shenzhen Medical Research Fund (nos. A2302011, A2502010, and A2503019), Shenzhen Science and Technology Program (JCYJ20230807111303007, RCBS20231211090624039, and JCYJ20250604142904006).

## Author contributions

Z.L., writing – original draft; X.X., data curation; C.C., data curation; Z.L., formal analysis; P.X., writing – review and editing; J.L., writing – review and editing; G.Y., writing – review and editing.

## Declaration of interests

The authors declare no competing interests.

## Declaration of generative AI and AI-assisted technologies in the manuscript preparation process

During the preparation of this work, the authors used Deepseek V4 pro in order to edit the language and polish the manuscript. After using this tool or service, the authors reviewed and edited the content as needed and take full responsibility for the content of the publication.

## STAR★Methods

### Key resources table

**Table:** 

REAGENT or RESOURCE	SOURCE	IDENTIFIER
**Antibodies**		

Anti-L-Lactyl Lysine Rabbit mAb	PTM BIO	Cat# PTM-1401RM; RRID: AB_2942013
Anti-L-Lactyl-Histone H3 (Lys9)	PTM BIO	Cat# PTM-1419RM; RRID: AB_3076695
Anti-L-Lactyl-Histone H3 (Lys18)	PTM BIO	Cat# PTM-1406RM; RRID: AB_2909438
Anti-L-Lactyl-Histone H4 (Lys8)	PTM BIO	Cat# PTM-1415RM; RRID: AB_3101829
Anti-L-Lactyl-Histone H4 (Lys12)	PTM BIO	Cat# PTM-1411RM; RRID: AB_2941896
Anti-Histone H3 Rabbit mAb (CT)	PTM BIO	Cat# PTM-1002RM; RRID: AB_3718014
Anti-Histone H4 Rabbit mAb (CT)	PTM BIO	Cat# PTM-1015RM; RRID: AB_3101866
ANTI-CXCL1/GRO ALPHA (MOUSE)	Abcam	#ab322200
ANTI-CXCL1/GRO ALPHA (HUMAN)	Abcam	Cat# ab89318; RRID: AB_2049712
ANTI-PDGFR ALPHA ANTIBODY	Abcam	Cat# ab203491; RRID: AB_2892065
β-ACTIN	CST	#4967S
Goat anti-mouse IgG-HRP	BOSTER	Cat# BA1050; RRID: AB_2904507
Goat anti-rabbit IgG-HRP	BOSTER	Cat# BA1054; RRID: AB_2734136
FITC ANTI-MOUSE CD14 ANTIBODY	BioLegend	Cat# 123307; RRID: AB_940578
FITC ANTI-MOUSE LY-6G ANTIBODY	BioLegend	Cat# 127605; RRID: AB_1236488

**Biological samples**		

Human synovial tissue	The Eighth Affiliated Hospital of Sun Yat-Sen University	#2024-236-02
Mouse synovial tissue	Institutional Animal Care and Use Committee of Sun Yat-Sen University	#2024002365

**Chemicals, peptides, and recombinant proteins**		

chicken type II collagen	Chondrex	#20012
Freund’s complete adjuvant	Chondrex	#7001
Freund’s incomplete adjuvant	Chondrex	#7002
curdlan	Wako Pure Chemical Industries	#034-23311
Stiripentol	MCE	#HY-103392
GNE140	MCE	#HY-100742A
collagenase type II	Gibco	#17101015
recombinant CXCL1 protein	SinoBiological	50150-MNCE
Lipofectamine RNAiMAX	Invitrogen	#13778150
TRIzol reagent	Invitrogen	#15596-026
Evo M-MLV RT Master Mix	AG	#11706
protease inhibitor	CWBIO	#CW2200S
RIPA buffer	Sigma-Aldrich	#R0278
phosphatase inhibitors	CWBIO	#CW2383S
hematoxylin	Servicebio	#G1004
eosin	BOSTER	#AR1180-2

**Critical commercial assays**		

Mouse CXCL1 ELISA Kit	Abcam	#ab216951
Proteome Profiler Mouse Chemokine Array Kit	R&D Systems	#ARY020
tartrate-resistant acid phosphatase staining kit	Sigma	#387A
SP Rabbit & Mouse HRP Kit	CWBIO	#CW2069S
BCA protein assay Kit	CWBIO	#CW0014S
SYBR Green Premix Pro Taq HS qPCR Kit	AG	#11718
Safranin O-Fast Green staining Kit	Solarbio,	#G1371
NovoNGS CUT&Tag 3.0 High-Sensitivity Kit	NovoProtein	#N259-YH01

**Deposited data**		

Serum Metabolomics data	EMBL-EBI	MTBLS5297
Microarray data	GEO	GSE36700
RNA-seq data	GEO	GSE210887
RNA-seq data	GEO	GSE276556
CUT&Tag-seq data	GEO	GSE327688

**Experimental models: Organisms/strains**		

DBA/1 male mice	GemPharmatech	N/A
SKG mice	Shanghai Model Organism Center	N/A

**Oligonucleotides**		

Primers for RT-qPCR assay, see Table S3	This paper	N/A
Primers for CUT&Tag-qPCR assay, see Table S5	This paper	N/A

**Software and algorithms**		

ImageJ	Schneider et al.	https://imagej.net/software/imagej/
Bowtie2	Langmead and Salzberg	https://bowtie-bio.sourceforge.net/bowtie2/index.shtml
Samtools	Li et al.	https://samtools.sourceforge.net/
TrimGalore	Krueger F	https://www.bioinformatics.babraham.ac.uk/projects/trim_galore/
GraphPad Prism 8.0	GraphPad Software	https://www.graphpad.com/
RadiAnt DICOM Viewer software	Medixant	https://www.radiantviewer.com/

### Experimental model and study participant details

#### Human samples

Synovial tissues were obtained from OA and RA patients undergoing total knee replacement at the orthopedic department of the Eighth Affiliated Hospital of Sun Yat-Sen University. The study included 6 patients with OA (59.3±8.5; 3 women and 3 men) and 6 patients with RA (56.9±7.5; 3 women and 3 men). All participants provided informed consent, and the study was approved by the hospital’s Ethics Committee: 2024-236-02. RA patients were diagnosed according to the 2010 classification criteria established by the American College of Rheumatology (ACR) and the European League Against Rheumatism (EULAR). Detailed patient characteristics for the OA and RA groups are presented in Table S1.

#### Mice

DBA/1J mice were purchased from GemPharmatech, and SKG (ZAP-70 W163C mutation) mice were kindly provided by the Shanghai Model Organism Center, Inc. (Shanghai, China). Male DBA/1J mice were used for the CIA model, and female BALB/c mice were used for the SKG model. These sex-specific selections follow the established standards in the field. All mice were housed and bred under pathogen-free conditions, and age-matched animals were used for experiments. The numbers of independent mice analyzed for each outcome are specified in the figure legends. For animal experiments requiring anesthesia, Tribromoethanol is administered at a concentration of 1.25% via intraperitoneal injection at a dosage of 200 mg/kg. The method of euthanasia for experimental animals was cervical dislocation after anesthesia. All animal procedures were conducted in accordance with protocols approved by the Institutional Animal Care and Use Committee of Sun Yat-Sen University: 2024002365.

#### Collagen-induced arthritis (CIA) model

To establish the CIA model, DBA/1 male mice (6–8 weeks old, 20–30 g) received a subcutaneous injection of 100 μL of a homogenized emulsion containing chicken type II collagen (Chondrex, #20012) mixed with an equal volume of Freund’s complete adjuvant (Chondrex, #7001) on day 1. On day 21, a booster immunization was administered using 100 μL of an emulsion prepared with chicken type II collagen and Freund’s incomplete adjuvant (Chondrex, #7002). The injection was performed slowly into the tail, carefully avoiding regions with enlarged blood vessels from the initial injection.

Arthritis severity in each limb was scored as follows: 0, normal; 1, redness or inflammation in a single joint; 2, redness or inflammation in multiple joints; 3, redness or inflammation across the entire paw; and 4, ankylosis or deformity. The total arthritis score for each mouse was determined by summing the scores for all four limbs. Hind paw thickness was measured with a vernier caliper to assess inflammation severity.

#### SKG arthritis model

To induce arthritis in SKG mice, 8-week-old female SKG mice were intraperitoneally injected with 3 mg of curdlan on day 1. Arthritis severity in each limb was scored as follows: 0, no swelling or redness; 0.1, swelling or redness in a single digit; 0.5, mild swelling and/or redness in the wrist or ankle joint; and 1, severe swelling in a larger joint. The total arthritis score for each mouse was calculated as the sum of scores from all four limbs.

#### Drug administration *in vivo*

For the prevention and treatment of CIA, DBA/1J mice were administered either 1 mg/kg of anti-CXCL1 monoclonal antibody (mAb) (R&D Systems, #MAB453) or 200 μg of rabbit IgG control via intravenous injection one day before collagen immunization and one day before collagen boosting. GNE140 (MCE, #HY-100742A), formulated in 20% SBE-β-CD saline, was administered orally at a dose of 200 mg/kg daily from day 21 to day 48. Stiripentol (MCE, #HY-103392), also formulated in 20% SBE-β-CD saline, was administered orally at a dose of 150 mg/kg daily over the same period. For the prevention and treatment of SKG, mice were administered either 1 mg/kg of anti-CXCL1 monoclonal antibody (mAb) (R&D Systems, #MAB453) or 200 μg of rabbit IgG control via intravenous injection one day before induction and on day 12. GNE140 (MCE, #HY-100742A), formulated in 20% SBE-β-CD saline, was administered orally at a dose of 200 mg/kg daily from day 0 to day 24. Stiripentol, also formulated in 20% SBE-β-CD saline, was administered orally at a dose of 150 mg/kg daily over the same period. The dose of 150 mg/kg (daily, oral gavage) was based on published preclinical studies that established stiripentol as an LDHA inhibitor.

#### Primary cell cultures

Primary human FLS were isolated from the OA and RA synovial tissues described above. Primary mouse FLS were isolated from ankle synovial tissues of SKG or CIA mice, with donor characteristics described in the method details section. FLS were isolated by enzymatic digestion and plastic adherence and maintained in DMEM/F12 supplemented with 10% fetal bovine serum at 37°C in a humidified atmosphere containing 5% CO_2_. Cells at passages 3–6 were used for experiments. Bone marrow cells used in migration assays were isolated from the femurs and tibias of SKG or CIA mice. Each biological replicate corresponded to a distinct human donor or individual mouse. Multiple wells or cultures derived from the same donor or mouse were not counted as independent biological replicates. The number of biological replicates for each experiment is specified in the corresponding figure legend. Detailed isolation and assay procedures are provided in METHOD DETAILS.

### Method details

#### Data preprocessing and model construction

In the serum metabolomics data (MTBLS5297) derived from the study by Cheng et al., we analyzed 302 serum metabolites from 27 healthy controls (HF) and 76 RA patients.^36^ A hybrid analytical approach combining LASSO regression and random forest algorithms was employed. To avoid data leakage, the dataset was first split into training (70%) and test (30%) subsets stratified by group (HF vs. RA) using the “caret” R package. All feature selection and model development were performed exclusively on the training set, while the test set was held out until final model evaluation. LASSO regression was constructed on the training set using the “glmnet” R package, with optimal feature selection achieved through 5-fold cross-validation. The lambda.1se parameter identified 66 metabolically relevant metabolites. Subsequently, the random forest model was developed on the same training cohort using the “randomForest” R package, with variable importance quantified via the Gini importance using the varImpPlot function. The *p*-value for Gini importance was calculated through permutation tests using the “rfPermute” R package. Model validation was performed on the held-out test set, and predictive performance was evaluated using receiver operating characteristic (ROC) curve analysis implemented in the “pROC” R package.

#### Fibroblast-like synoviocytes (FLS) isolation and culture

Tissues were sectioned into 0.1 g fragments and transferred to 5 mL microtubes. The fragments were minced with scissors and digested enzymatically with 2 mL of 0.5% collagenase type II (Gibco, #17101015) for 60 minutes on a rotor at 37°C. Digestion was stopped by adding 8 mL of FLS medium, consisting of DMEM/F12 (Gibco) supplemented with 10% fetal bovine serum (Gibco). The partially dissociated cells were centrifuged at 400 × g for 5 minutes, and the supernatant was discarded. The cell pellet was resuspended in FLS medium, seeded onto plastic culture dishes, and incubated in a humidified environment with 1.5% O_2_ and 5% CO_2_. Nonadherent cells were removed after overnight incubation, and FLS from passages 3 to 6 were used for subsequent experiments.

#### Mouse synovial tissue cells and BM cells isolation

Synovial tissues from the ankle were digested in 1640 medium (Gibco, #C11875500BT) containing 0.5% type II collagenase at 37°C for 1 hour. Dispersed cells were collected by centrifugation at 1500 rpm for 5 minutes. For BM cell isolation, the femurs and tibias were dissected from adult mice and flushed with PBS containing 0.1% BSA to remove residual tissue debris. The resultant bone marrow cell suspensions were collected by centrifugation for 5 minutes at 1500 rpm, followed by re-suspension in pre-cooled complete medium.

#### BM cells migration assay

BM cells migration assays were conducted using polycarbonate membrane Transwell inserts with 5.0-μm pores in a 24-well plate (Corning). A total of 5 × 10^4^ FLS in 600 μL of DMEM/F12 or cell-free culture supernatant were seeded into the lower chambers. After FLS adhesion, RNA interference treatments were applied or omitted as appropriate. The culture supernatant was then replaced with FBS-free medium, with or without recombinant CXCL1 protein (SinoBiological, 50150-MNCE) and anti-CXCL1 neutralizing antibody (R&D Systems, #MAB453). In the upper chambers, 1 × 10^6^ BM cells in 100 μL of FBS-containing culture medium were seeded. Following a 12-h incubation, the culture supernatant from the lower chambers was collected, and BM cells numbers were quantified by flow cytometry.

#### Flow cytometry

To evaluate the recruitment capacity of FLS, BM cells collected from the lower chambers of the Transwell system were analyzed using a BD FACSCelesta flow cytometer. For the assessment of Ly6G^+^ neutrophils and CD14^+^ monocytes in ankle tissues, synovial cells were isolated as previously described. The collected cells were stained with live/dead 780 dye (Invitrogen, #L34992) for 15 minutes, followed by incubation with anti-CD45-Pacific Blue (BioLegend, #103125) and either anti-Ly6G-FITC (BioLegend, #127605) or anti-CD14-FITC (BioLegend, #123307) for 30 minutes in the dark. Live Ly6G^+^ neutrophils and CD14^+^ monocytes were then detected using a BD FACSCelesta flow cytometer.

#### Measurement of lactate production

Lactate levels in synovial tissues were quantified by homogenizing the samples on ice in lysis buffer. The homogenates were centrifuged at 12,000 × g for 10 minutes at 4°C, and the resulting supernatants were collected. Lactate concentrations were measured using the L-Lactate Assay Kit (Abcam, #ab65330) according to the manufacturer’s instructions.

#### Measurement of CXCL1 secretion

CXCL1 levels in synovial fluid and culture supernatants were quantified as described above. CXCL1 concentrations were measured using the Mouse CXCL1 ELISA Kit (Abcam, #ab216951) according to the manufacturer’s instructions.

#### RNA interference

Gene knockdown was achieved using targeting and control siRNAs obtained from GenePharma. For transfection, 0.2 nmol of siRNA was mixed with 4 μL of Lipofectamine RNAiMAX (Invitrogen, #13778150) in 100 μL of Opti-MEM Reduced Serum Medium (Thermo Fisher, #31985070) and incubated separately at 37°C for 5 minutes. The siRNA-Lipofectamine mixtures were then combined and incubated for an additional 20 minutes at 37°C before being applied to target cells. Cells were maintained in reduced-serum Opti-MEM for 6 hours post-transfection, after which the medium was replaced with DMEM containing 10% FBS. Gene silencing efficiency was assessed three days later, and the cells were subsequently used for further experiments. Control experiments included nontargeting siRNA. The siRNA sequences used are provided in Table S2.

#### RNA preparation and RT‒qPCR

Total RNA was extracted from cells using TRIzol reagent (Invitrogen, #15596-026) and reverse transcribed into cDNA using the Evo M-MLV RT Master Mix (AG, #11706) according to the manufacturer’s instructions. Reverse transcription quantitative PCR (RT-qPCR) was performed using the SYBR Green Premix Pro Taq HS qPCR Kit (AG, #11718). Gene expression levels were quantified using the 2^-ΔΔCt^ method and normalized to β-actin. Primer sequences for the amplification of target genes are provided in Table S3.

#### Protein extraction and western blot analysis

Cells were harvested and lyzed in RIPA buffer (Sigma-Aldrich, #R0278) supplemented with 1% protease inhibitor (CWBIO, #CW2200S) and phosphatase inhibitors (CWBIO, #CW2383S) for 30 minutes on ice. The lysates were clarified by centrifugation at 14,000 rpm for 10 minutes at 4°C, and the supernatant was collected. Protein concentrations were measured using a BCA protein assay kit (CWBIO, #CW0014S) according to the manufacturer’s protocol. Samples were prepared with Beyotime loading buffer (#P0015), boiled, and subjected to western blot analysis. Proteins were separated by SDS-PAGE and transferred to PVDF membranes (Millipore, #IPVH00010). Membranes were blocked with 5% BSA, incubated with primary antibodies overnight at 4°C, and then with HRP-conjugated secondary antibodies for 1 hour at room temperature. Protein detection was performed using enhanced chemiluminescence (ECL) (Millipore, #WBKLS0500). Details of the antibodies used are provided in Table S4. The integrated density of each band was measured using ImageJ (NIH). The signal intensity of each target protein was normalized to that of the corresponding loading control (β-actin, histone H3, or histone H4) detected on the same membrane after stripping and re-probing.

#### Cytokine array assay

FLS were seeded into 12-well plates at a density of 1 × 10^5^ cells per well. After stimulation with sodium lactate for 3 days, culture supernatants were collected. The collected supernatants were analyzed using the Proteome Profiler Mouse Chemokine Array Kit (R&D Systems, #ARY020) following the manufacturer’s instructions.

#### Micro-CT scanning

Mouse ankle joints were harvested and fixed in 4% paraformaldehyde. Micro-CT scans were conducted using a Siemens Inveon CT scanner, and three-dimensional reconstructions were generated with RadiAnt DICOM Viewer software (version 5.0.2).

#### Histological staining

Samples were fixed in 4% paraformaldehyde for 24 hours, and bone-containing specimens were decalcified in EDTA solution for 14 days before paraffin embedding. Tissue sections were rehydrated by sequential immersion in xylene and a graded ethanol series.

#### H&E staining

Rehydrated tissue sections were stained with hematoxylin (Servicebio, #G1004) for 10 minutes, followed by a brief immersion in 1% alcoholic hydrochloric acid for 5 seconds and rinsing with PBS. The sections were then counterstained with eosin (BOSTER, #AR1180-2) for 2 minutes. After washing, the sections were dehydrated and mounted with neutral resin. Imaging was performed using a standard microscope. Arthritis inflammation was evaluated on H&E-stained sections using a graded scoring system: 0, normal joint; 1, mild thickening of the lining layer or mild inflammatory cell infiltration; 2, both mild thickening and inflammatory cell infiltration; 3, moderate thickening and inflammatory cell infiltration with cells present in the synovial space; and 4, severe inflammatory cell infiltration in the synovium.

#### Safranin O-Fast Green staining

Safranin O-Fast Green staining was performed using a modified kit (Solarbio, #G1371). Rehydrated sections were stained with Weigert’s iron hematoxylin for 5 minutes, followed by differentiation in an acidic solution for 15 seconds. Sections were then stained with Fast Green for 5 minutes, followed by brief differentiation in an acidic solution for 10 seconds. After air-drying, the sections were counterstained with Safranin O for 5 minutes, dehydrated, and mounted with neutral resin. Imaging was conducted using a microscope. Cartilage damage was evaluated on stained sections using a graded scoring system: 0, normal joint; 1, partial erosion of the cartilage surface; 2, erosion with cartilage destruction; and 3, extensive cartilage erosion and destruction covered by connective tissue.

#### Tartrate-resistant acid phosphatase staining

Rehydrated sections were stained with the tartrate-resistant acid phosphatase staining kit (Sigma, St. Louis, MO, USA) according to the manufacturer’s protocol. TRAP-positive cells were considered osteoclasts.

#### Tissue immunofluorescence staining

Samples were fixed in 4% paraformaldehyde for 24 hours, decalcified in 50 mM EDTA for two weeks, and embedded in paraffin. Sections were deparaffinized, rehydrated, and permeabilized with 0.5% Triton X-100 for 30 minutes. Antigen retrieval was performed by heating the sections in 10 mM citrate buffer in a microwave at 750 W for 20 minutes. After cooling to room temperature, sections were blocked with 5% normal goat serum and incubated overnight at 4°C with primary antibodies. The following day, sections were washed with PBS and incubated with fluorescein-labeled secondary antibodies for 1 hour at room temperature in the dark. Confocal images were captured using a Nikon Eclipse Ni-E system and the primary antibodies used are detailed in Table S4.

#### Tissue immunohistochemical staining

Following established sample preparation protocols, sections were deparaffinized, rehydrated, and subjected to antigen retrieval by microwaving in 10 mM citrate buffer at 750 W for 20 minutes. After cooling to room temperature, sections were treated with 3% H_2_O_2_ for 25 minutes to quench endogenous peroxidase activity and blocked with 5% normal goat serum for 30 minutes. Primary antibodies were applied, and sections were incubated overnight at 4°C. The following day, secondary antibodies were applied, and detection was performed using the SP Rabbit & Mouse HRP Kit (CWBIO, #CW2069S). Imaging was conducted using a microscope, and the primary antibodies used are detailed in Table S4.

#### CUT&Tag assay, qPCR, and sequencing

##### CUT&Tag assay

The CUT&Tag assay was performed using the NovoNGS CUT&Tag 3.0 High-Sensitivity Kit (NovoProtein, #N259-YH01). Fibroblast-like synoviocytes (FLS, 1 × 10^5^) were washed twice with 0.5 mL of wash buffer, mixed with ConA beads, and incubated overnight at 4°C with one of the following primary antibodies: anti-L-Lactyl-Histone H3 (Lys9) (PTM BIO, #PTM-1419RM), anti-L-Lactyl-Histone H3 (Lys18) (PTM BIO, #PTM-1406RM), anti-L-Lactyl-Histone H4 (Lys8) (PTM BIO, #PTM-1415RM), or anti-L-Lactyl-Histone H4 (Lys12) (PTM BIO, #PTM-1411RM). Samples were then incubated with goat anti-rabbit IgG antibody (NovoProtein, 1:500) for 1 hour at room temperature (RT). After washing, cells were incubated with pAG-Tn5 for 1 hour at RT, and tagmentation was activated with MgCl_2_ for 1 hour at 37°C. DNA was isolated using NovoNGS DNA Extract Beads and dissolved in TE buffer. Sequencing-ready libraries were generated by PCR amplification with N5 and N7 primers, followed by purification with NovoNGS DNA Clean Beads. Library quality was assessed using an Agilent Bioanalyzer 2100 system.

##### CUT&Tag-qPCR

A 10-fold dilution of the purified DNA from the CUT&Tag assay was used for CUT&Tag-qPCR analysis, with primer sequences listed in Table S5.

##### CUT&Tag sequencing

Index-coded samples were clustered using the cBot Cluster Generation System with the TruSeq PE Cluster Kit v3-cBot-HS (Illumina) according to the manufacturer’s protocol. Libraries were sequenced on the Illumina NovaSeq platform at Novogene Science and Technology (Beijing, China), generating 150 bp paired-end reads.

##### CUT&Tag data analysis

CUT&Tag data analysis was performed with slight modifications to established protocols. Raw reads were filtered and trimmed to remove adaptor sequences and low-quality reads using TrimGalore (version 0.6.6) with the parameters -q 20 –phred33 –stringency 3. Clean reads were aligned to the mouse reference genome mm10 (GRCm38) using Bowtie2 (version 2.5.1) with the parameters -p 6 -q, retaining only uniquely mapped reads. Peak calling was conducted using MACS2 with the parameters -q 0.05 --call-summits --nomodel --shift 100 --extsize 200 --keep-dup all. Aligned and deduplicated BAM files were used for peak simulations without additional filtering. CUT&Tag-seq data were normalized as reads per kilobase per million (RPKM) using the bamCoverage command, and heatmaps were generated using the computeMatrix and plotHeatmap commands in deepTools (version 2.3.6.0). The normalized data were visualized with the Integrative Genomics Viewer (IGV).

### Quantification and statistical analysis

#### Statistical analysis

Data are presented as scatterplots with means ± standard deviations. Statistical analyses were performed using GraphPad Prism 8.0 (GraphPad Prism Software, CA, USA). Metabolomics analyses were performed in R (version 4.4.2) using the caret, glmnet, randomForest, rfPermute, and pROC packages, as described in “Data preprocessing and model construction”. Sample normality was assessed with the Shapiro‒Wilk test. Differences between independent samples were analyzed using two-tailed Student’s t-tests, while paired samples were evaluated with paired t-tests. A two-way repeated-measures ANOVA was used to compare arthritis scores and hind paw thickness across groups. Sample sizes (n) are specified in the figure legends, and a *p*-value <0.05 was considered statistically significant. Statistical significance is indicated as follows: ∗*p* < 0.05, ∗∗*p* < 0.01, and ∗∗∗*p* < 0.001.

#### Experimental design and statistical rigor

Mice were randomly assigned to experimental groups using a random number table. Arthritis scoring and paw thickness measurements were performed by two independent investigators blinded to group allocation. Pre-established exclusion criteria included: (i) death or severe morbidity requiring early euthanasia, (ii) failure to develop arthritis in the control group, and (iii) technical failure during tissue processing or sample collection. Sample sizes were determined based on power analysis (G∗Power; α = 0.05, power = 0.80) using effect sizes estimated from pilot experiments and published literature, with a minimum of *n* = 6 mice per group for *in vivo* experiments. Each *in vitro* experiment was performed with at least three independent biological replicates, and each *in vivo* experiment was performed with at least two independent cohorts, as specified in the figure legends.

## Supplemental information

Document S1. Figures S1–S7 and Tables S1–S5

Data S1. Original western blot images supporting the immunoblot analyses presented in the main and supplemental figures, related to Figures 1–3 and Figures S1

Data S2. Source data underlying the quantitative analyses in this study

Data S3. Original instrument output for the CXCL1 ELISA analysis, related to Figure 3G

Data S4. Original instrument output for the Cxcl1 RT-qPCR analysis, related to Figure 3H
