# Supplementary material for: Histone lactylation in fibroblast-like synoviocytes drives CXCL1-mediated immune cell recruitment in rheumatoid arthritis
Source: iScience. 2026 Nov 4;29(11):118108. doi: 10.1016/j.isci.2026.118108 (PMC13756642; doi:10.1016/j.isci.2026.118108)
Supplement: Supplementary file 1 [file mmc1.pdf]

iScience, Volume *29*

Supplemental information

Histone lactylation in fibroblast-like synoviocytes drives CXCL1-mediated immune cell recruitment in rheumatoid arthritis

Zhidong Liu, Xiaojun Xu, Chaoqiang Chen, Zhexiao Lan, Peitao Xu, Jinteng Li, and Guiwen Ye

Document S1. Supplemental information, Figures S1–S7, and Tables S1–S5

Supplementary Figures

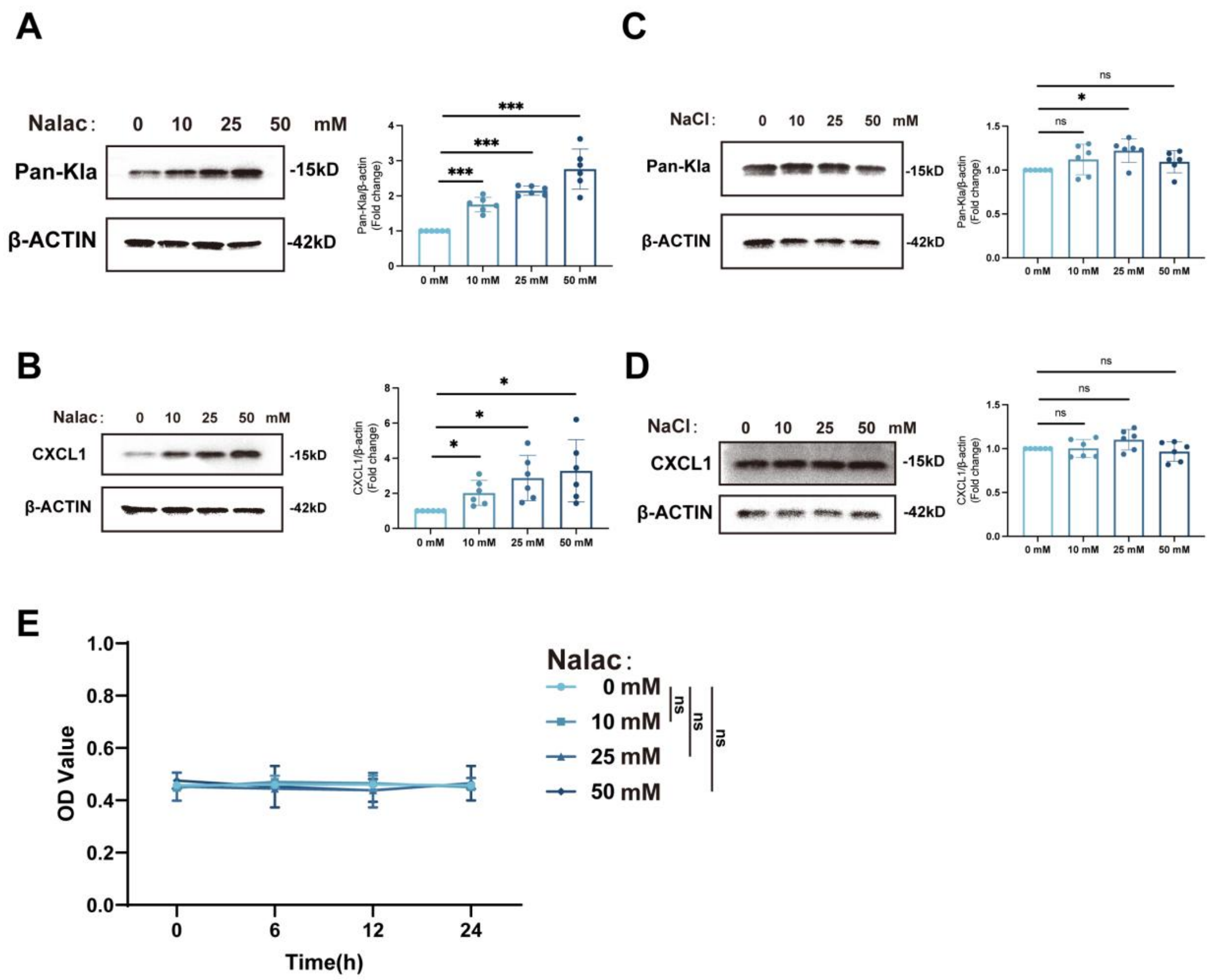

Figure S1 Related to Figure 2

(A) Western blot analysis showing levels of pan-Kla in FLS from SKG mice stimulated with different concentrations of sodium lactate for 24h (n=6 biological replicates). Parallel membranes were probed with the indicated antibodies using the same samples. Statistical analysis was performed using two-tailed unpaired t-tests.

(B) Western blot analysis showing levels of CXCL1 in FLS from SKG mice stimulated with different concentrations of sodium lactate for 24h (n=6 biological replicates). Parallel membranes were probed with the indicated antibodies using the same samples. Statistical analysis was performed using two-tailed unpaired t-tests.

(C) Western blot analysis showing levels of pan-Kla in FLS from SKG mice stimulated with different concentrations of sodium chloride for 24h (n=6 biological replicates). Parallel membranes were probed with the indicated antibodies using the same samples. Statistical analysis was performed using two-tailed unpaired t-tests.

(D) Western blot analysis showing levels of CXCL1 in FLS from SKG mice stimulated with different concentrations of sodium chloride for 24h (n=6 biological replicates). Parallel membranes were probed with the indicated antibodies using the same samples. Statistical analysis was performed using two-tailed unpaired t-tests.

(E) Cell viability of FLS treated with sodium lactate for 24 h was assessed by CCK-8 assay. The P value shown in the source data was based on a two-way ANOVA followed by Bonferroni's post hoc comparisons.

All data are presented as mean ± SD; *p < 0.05, **p < 0.01, ***p < 0.001.

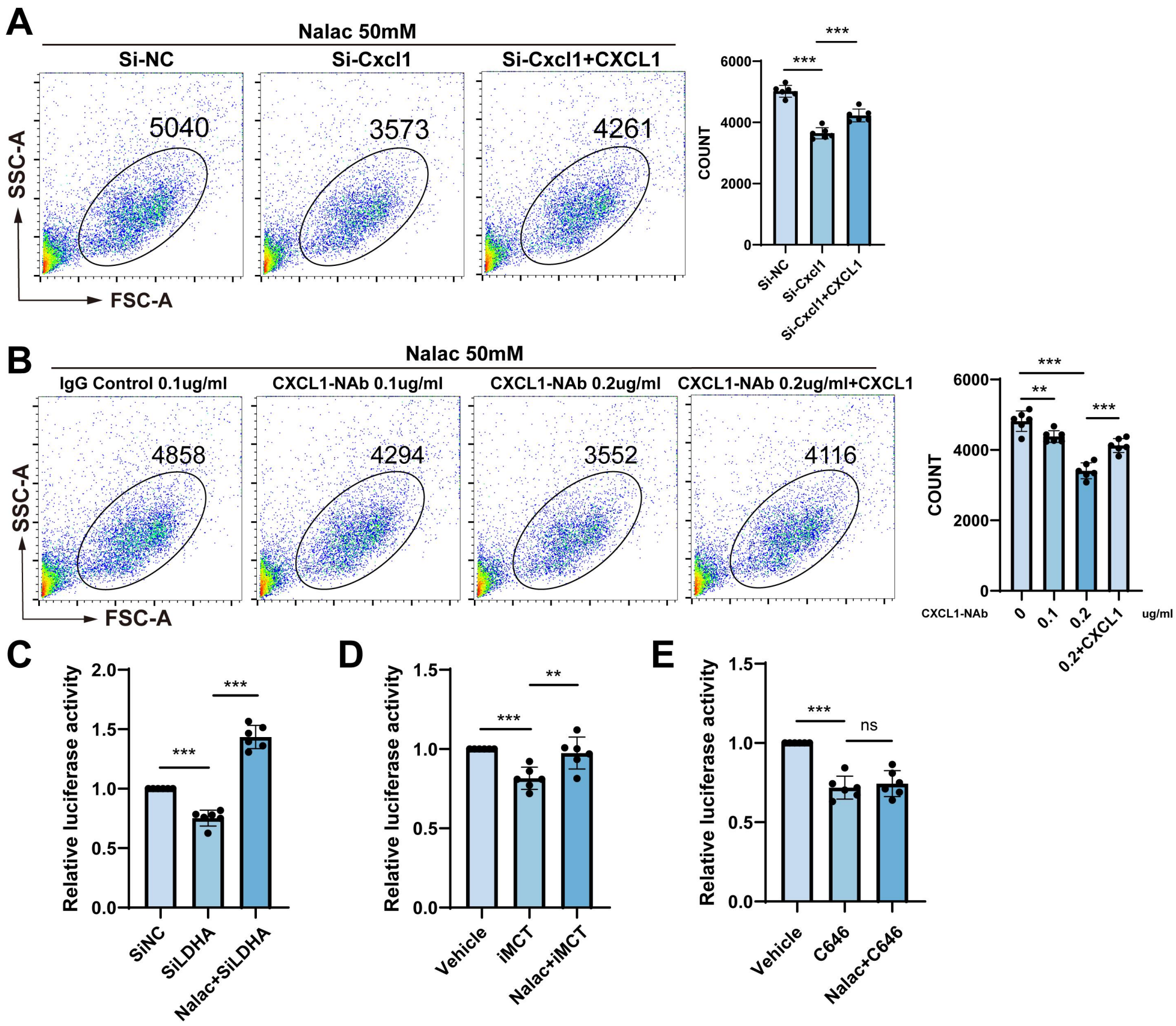

Figure S2 Related to Figure 3

(A) The number of BM cells recruited by FLS from Si-NC group and Si-Cxcl1 group with or without recombinant CXCL1 (50 ng/mL) supplementation in transwell assay via flow cytometric analysis (n=6 biological replicates). Statistical analysis was performed using two-tailed unpaired t-tests.

(B) The number of BM cells recruited by FLS from Cxcl1 NAb group(0.1ug/ml and 0.2ug/ml) and IgG group(0.1ug/ml) with or without recombinant CXCL1 (50 ng/mL) supplementation in transwell assay via flow cytometric analysis (n=6 biological replicates). Statistical analysis was performed using two-tailed unpaired t-tests.

(C) Cxcl1 promoter activity in FLS transfected with control or LDHA siRNA in the presence or absence of sodium lactate (n=6 biological replicates). Statistical analysis was performed using two-tailed unpaired t-tests.

(D) Cxcl1 promoter activity in FLS stimulated with 7ACC2 in the presence or absence of sodium lactate (n=6 biological replicates). Statistical analysis was performed using two-tailed unpaired t-tests.

(E) Cxcl1 promoter activity in FLS stimulated with C646 in the presence or absence of sodium lactate (n=6 biological replicates). Statistical analysis was performed using two-tailed unpaired t-tests.

All data are presented as mean ± SD; *p < 0.05, **p < 0.01, ***p < 0.001.

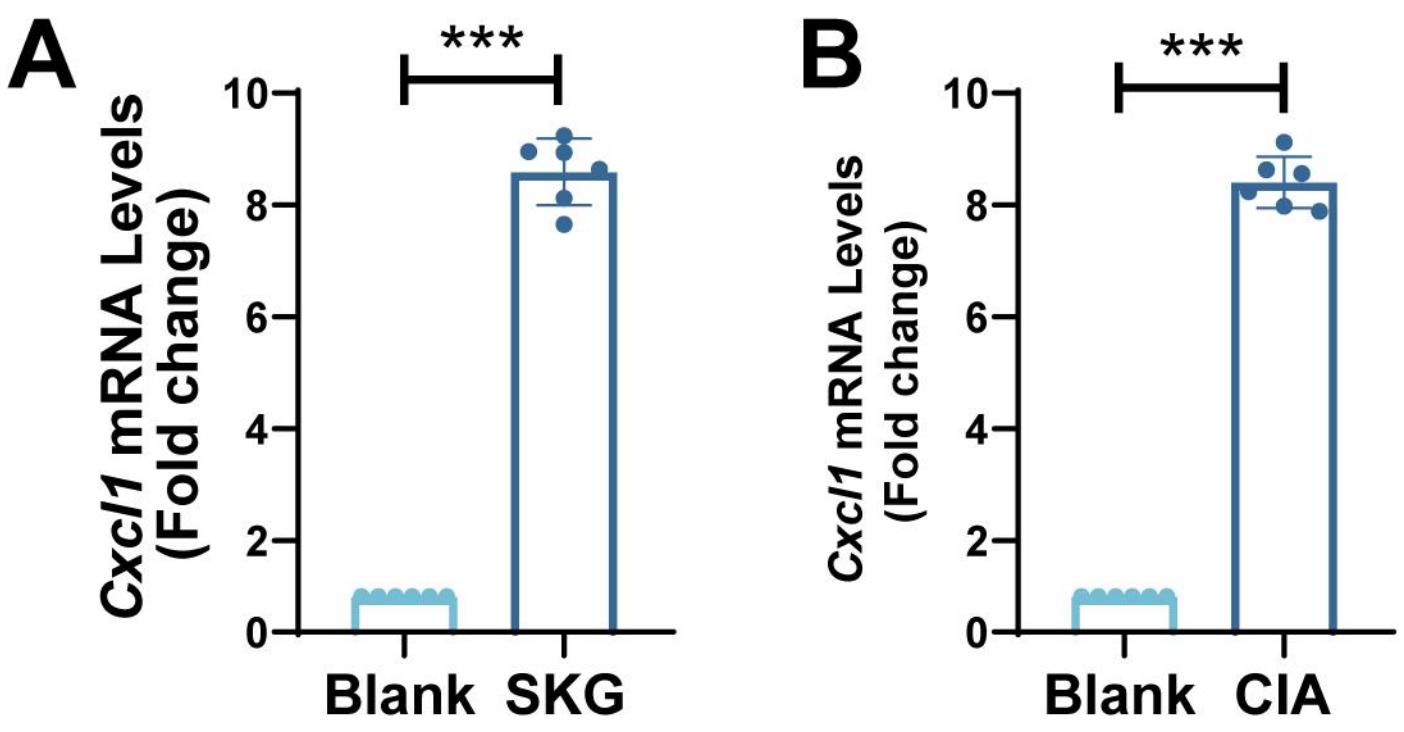

Figure S3 Related to Figure 4

(A) Synovial *Cxcl1* RNA levels in SKG mice compared to the Blank groups (n=6 biological replicates). Statistical analysis was performed using two-tailed unpaired t-tests.

(B) Synovial *Cxcl1* RNA levels in CIA mice compared to the Blank groups (n=6 biological replicates). Statistical analysis was performed using two-tailed unpaired t-tests.

All data are presented as mean ± SD; *p < 0.05, **p < 0.01, ***p < 0.001.

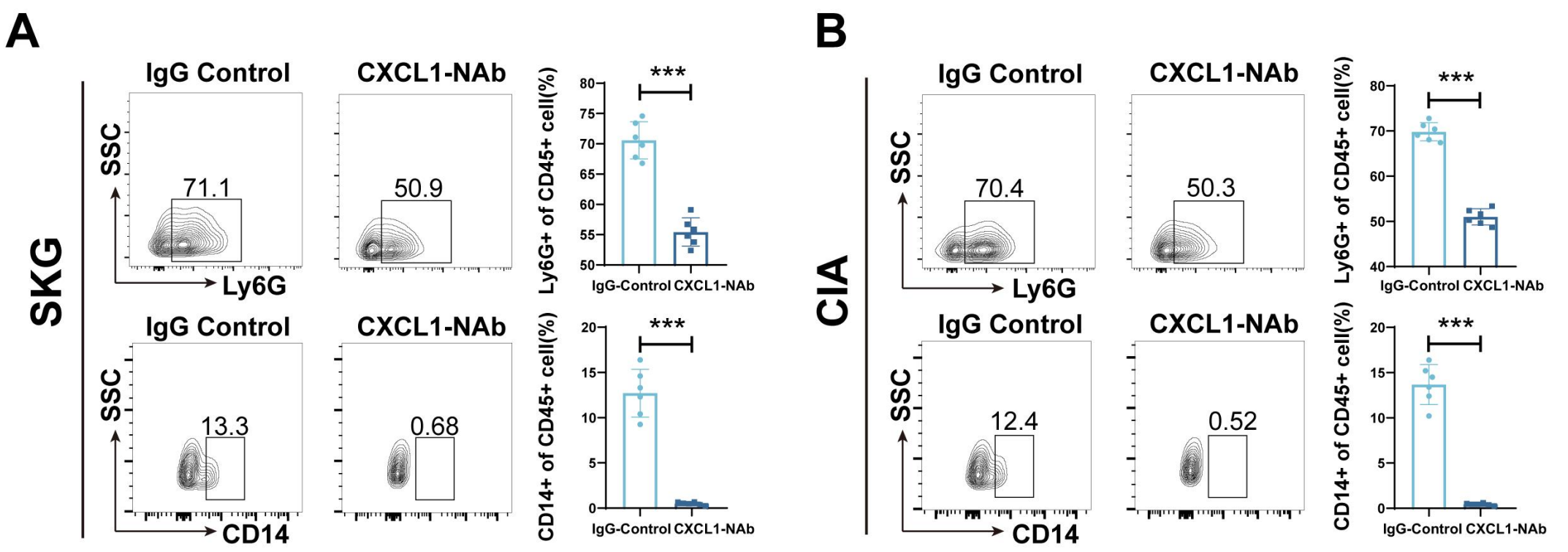

Figure S4 Related to Figure 5

(A) Flow cytometry assay showing Ly6G+ and CD14+ cell ratios in the synovial tissue of SKG mice treated with CXCL1 NAb or IgG (n=6 biological replicates). Statistical analysis was performed using two-tailed unpaired t-tests.

(B) Flow cytometry assay showing Ly6G+ and CD14+ cell ratios in the synovial tissue of CIA mice treated with CXCL1 NAb or IgG (n=6 biological replicates). Statistical analysis was performed using two-tailed unpaired t-tests.

All data are presented as mean ± SD; *p < 0.05, **p < 0.01, ***p < 0.001.

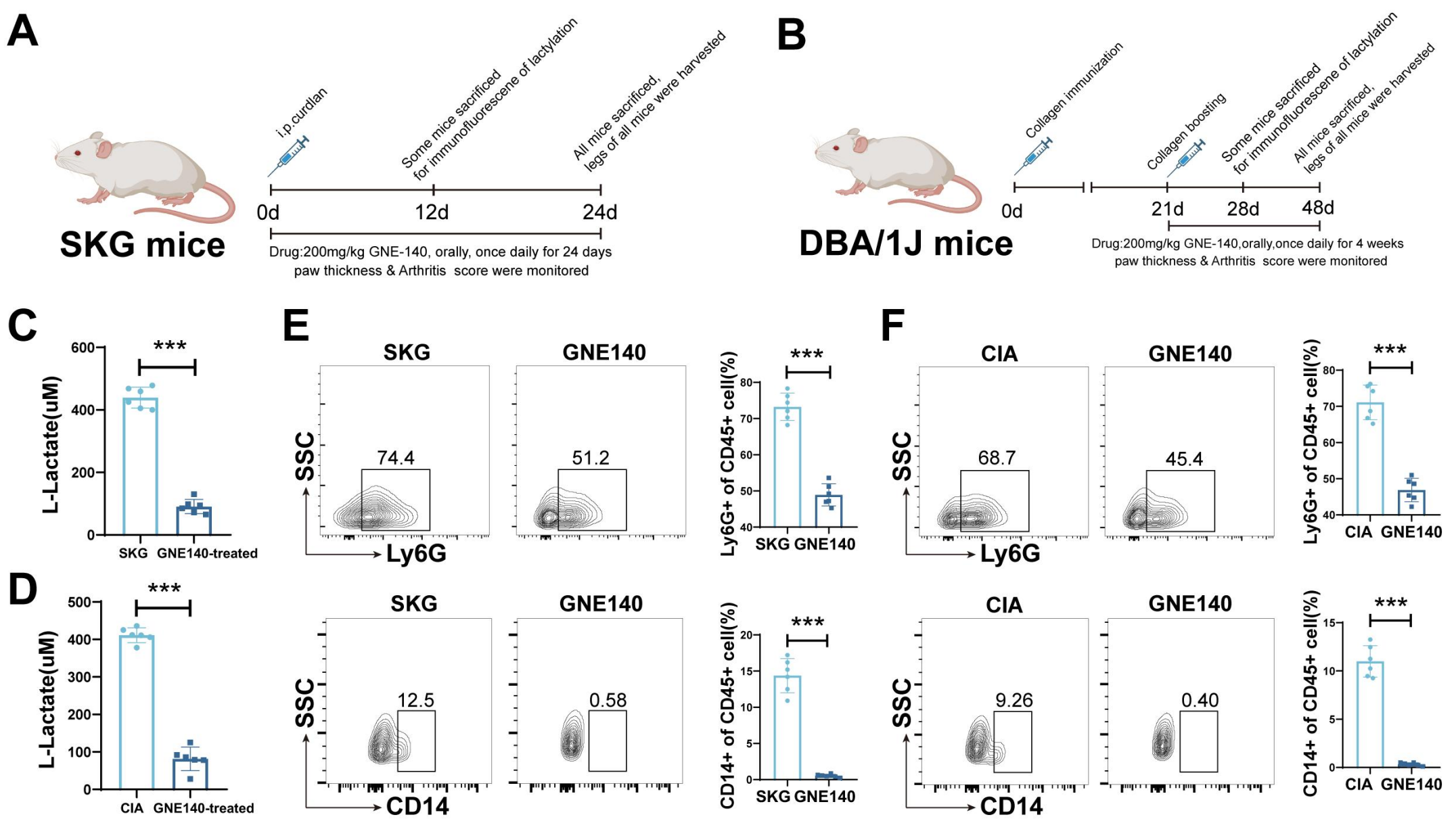

Figure S5 Related to Figure 6

(A) Schematic of SKG induction and GNE140 administration.

(B) Schematic of CIA induction and GNE140 administration.

(C) Lactate levels of ankle synovial tissues of SKG treated with GNE140 or vehicle were presented (n=6 biological replicates). Statistical analysis was performed using two-tailed unpaired t-tests.

(D) Lactate levels of ankle synovial tissues of CIA treated with GNE140 or vehicle were presented (n=6 biological replicates). Statistical analysis was performed using two-tailed unpaired t-tests.

(E) Flow cytometry assay showing Ly6G+ and CD14+ cell ratios in the synovial tissue of SKG mice treated with GNE140 or vehicle (n=6 biological replicates). Statistical analysis was performed using two-tailed unpaired t-tests.

(F) Flow cytometry assay showing Ly6G+ and CD14+ cell ratios in the synovial tissue of CIA mice treated with GNE140 or vehicle (n=6 biological replicates). Statistical analysis was performed using two-tailed unpaired t-tests.

All data are presented as mean ± SD; $^{*}p < 0.05$, $^{**}p < 0.01$, $^{***}p < 0.001$.

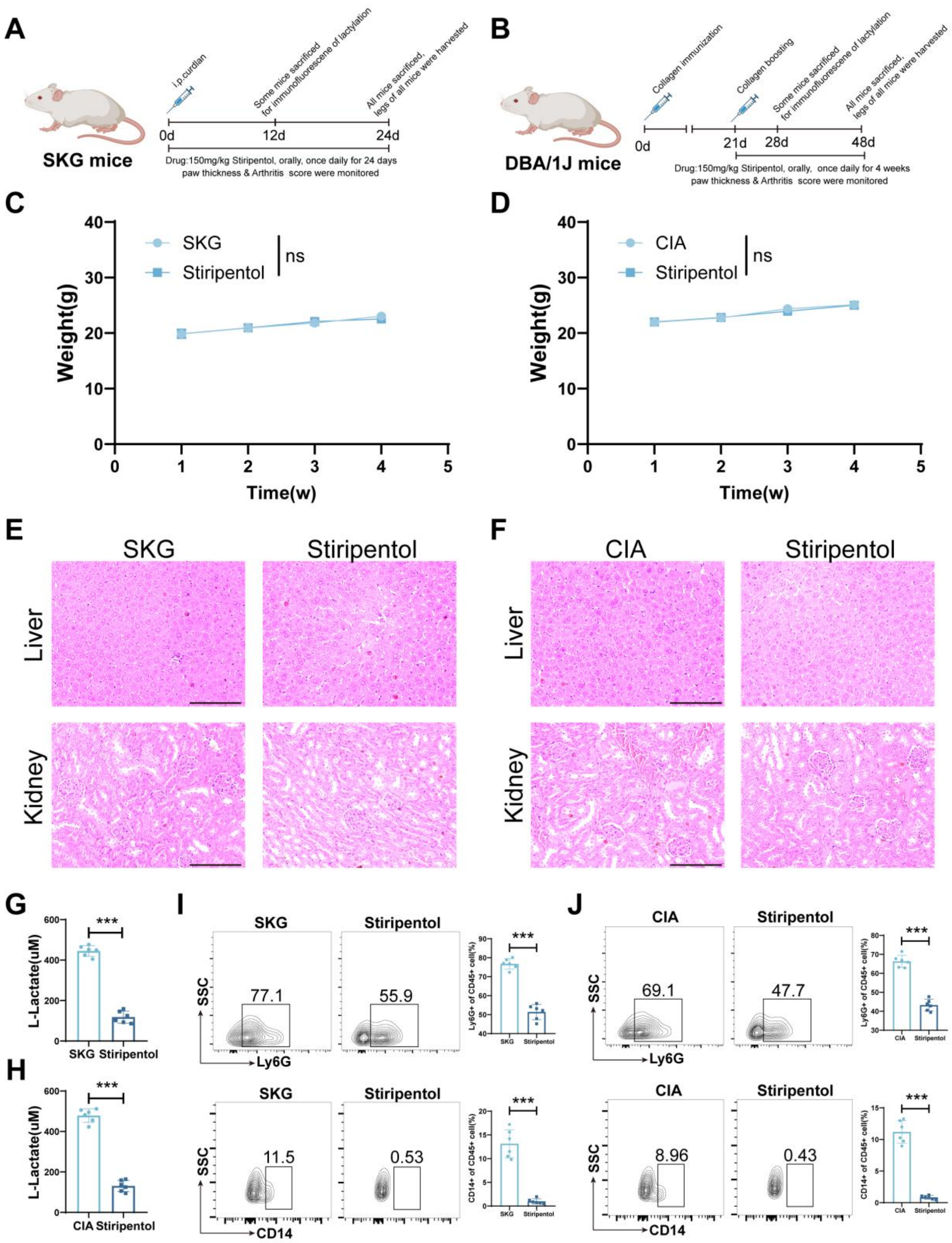

Figure S6 Related to Figure 7

(A) Schematic of SKG induction and Stiripentol administration.

(B) Schematic of CIA induction and Stiripentol administration.

(C) Body weight change in SKG mice over four weeks following stiripentol treatment. P value shown in source data was based on two-way ANOVA followed by Bonferroni's post hoc comparisons. (n=6 biological replicates).

(D) Body weight change in CIA mice over four weeks following stiripentol treatment. P value shown in source data was based on two-way ANOVA followed by Bonferroni's post hoc comparisons. (n=6 biological replicates).

(E) HE staining of liver and kidney from SKG mice in the vehicle and stiripentol-treated groups. Scale bar = 100 μm. (n=3 biological replicates).

(F) HE staining of liver and kidney from CIA mice in the vehicle and stiripentol-treated groups. Scale bar = 100 μm. (n=6 biological replicates).

(G) Lactate levels of ankle synovial tissues of SKG treated with Stiripentol or vehicle were presented (n=6 biological replicates). Statistical analysis was performed using two-tailed unpaired t-tests.

(H) Lactate levels of ankle synovial tissues of CIA treated with Stiripentol or vehicle were presented (n=6 biological replicates). Statistical analysis was performed using two-tailed unpaired t-tests.

(I) Flow cytometry assay showing Ly6G+ and CD14+ cell ratios in the synovial tissue of SKG mice treated with Stiripentol or vehicle (n=6 biological replicates). Statistical analysis was performed using two-tailed unpaired t-tests.

(J) Flow cytometry assay showing Ly6G+ and CD14+ cell ratios in the synovial tissue of CIA mice treated with Stiripentol or vehicle (n=6 biological replicates). Statistical analysis was performed using two-tailed unpaired t-tests.

All data are presented as mean ± SD; *p < 0.05, **p < 0.01, ***p < 0.001.

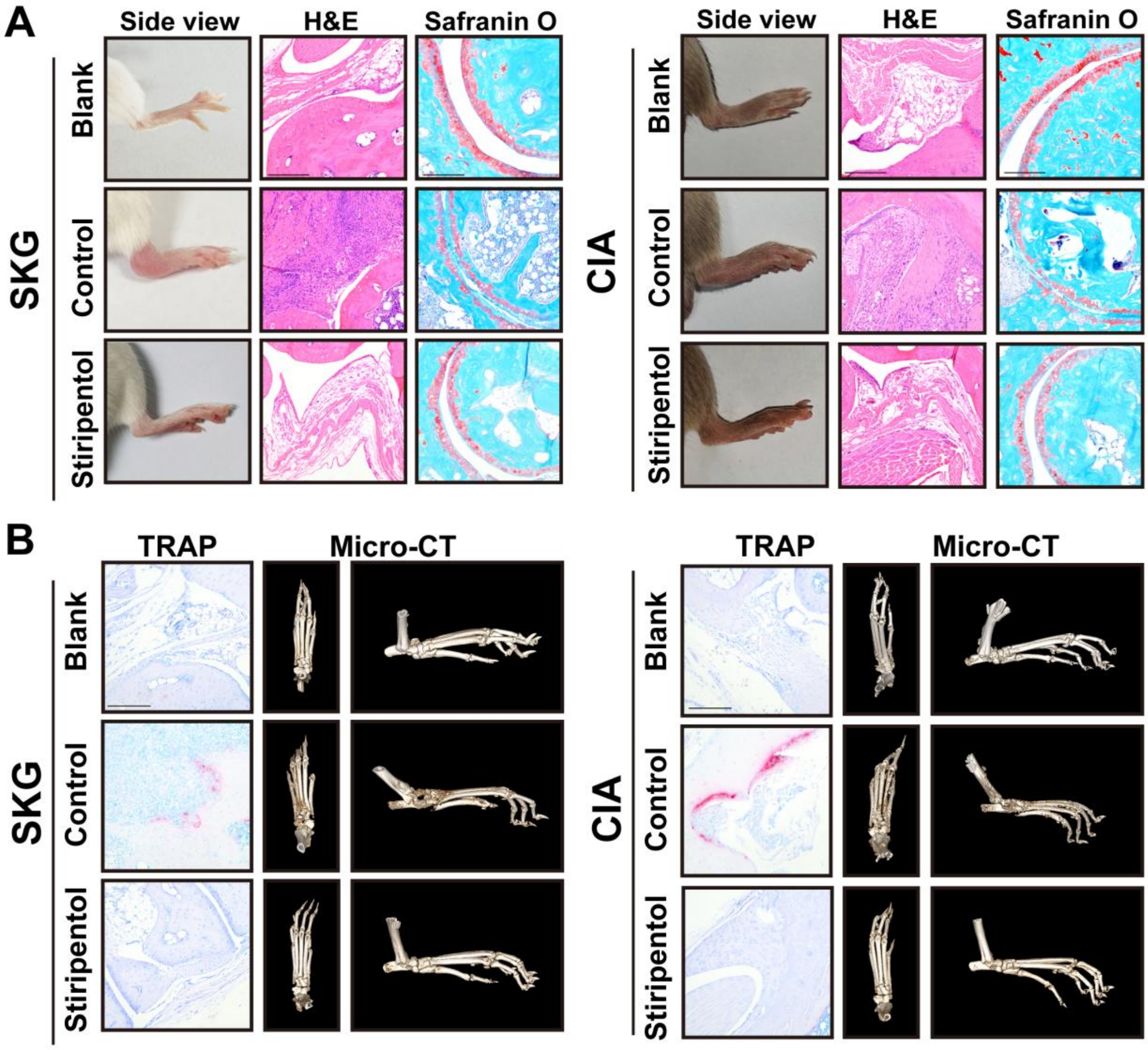

Figure S7 Related to Figure 7

(A) Macroscopic images, H&E staining and Safranin O staining of the ankle joints from SKG and CIA mice treated with Stiripentol or vehicle (n=3 biological replicates). Scale bar = 400 μm.

(B) TRAP staining and micro-CT images of the ankle joints from SKG and CIA mice treated with Stiripentol or vehicle (n = 3 biological replicates). Scale bar = 400 μm.

Supplementary Tables

Table S1. Characteristics of the OA and RA patients

	OA patients	RA patients
Number	6	6
Age, year	59.3±8.5	56.9±7.5
Sex, male/female	3/3	3/3
Disease duration, year	8.9±3.6	11.0±4.2
DAS28	-	5.3±1.3
CRP, mg/L	2.1±4.3	17.9±12.6*
ESR, mm/h	23.5±19.2	46.9±12.3*
RF, IU/ml	-	213.2±138.8
ACPA, U/ml	-	756.2±327.4
Anti-CCP ab, U/ml	-	1078.3±236.6
Medications		
csDMARDs, n(%)		
Methotrexate	0/6(0)	4/6(66.7)
Leflunomide	0/6(0)	2/6(33.3)
Glucocorticoids, n(%)		
Prednisone use	0/6(0)	4/6(66.7)
Dose	-	6.3±2.5
NSAIDs, n(%)	4/6(66.7)	2/6(33.3)
bDMARDs, n(%)	0/6(0)	0/6(0)

Mean±SD. OA, osteoarthritis; RA, rheumatoid arthritis; CRP, C reactive protein; ESR, erythrocyte sedimentation rate; DAS28, Disease Activity Score in 28 joints; RF, rheumatoid factors; ACPA, anti-citrullinated protein antibody; Anti-CCP ab, anti-cyclic citrullinated peptide antibody; csDMARDs, conventional synthetic disease-modifying antirheumatic drugs; NSAIDs,

non-steroidal anti-inflammatory drugs; bDMARDs, biologic disease-modifying antirheumatic drugs; SD, standard deviation. Data were analyzed using two-tailed Student's t tests; * indicates P< 0.05 compared to OA; ** indicates P< 0.01 compared to OA.

Table S2. Sequences of siRNA used in RNA Interference assay

siRNA		Sequence
Cxcl1	Forward	5'-GCAGACCAUGGCUGGGAUUTT-3'
	Reverse	5'-AAUCCCAGCCAUGGUCUGCTT-3'

Table S3. Sequences of primers used in RT-qPCR assay

Gene		Sequence
CXCL1	Forward	5'-CTGGGATTCACCTCAAGAACATC-3'
	Reverse	5'-CAGGGTCAAGGCAAGCCTC-3'
Cxcl1	Forward	5'-GGGAGGAGCGATGTCCTTTC-3'
	Reverse	5'-GAAGACTCTTGGTCAGCGGG-3'
β-ACTIN	Forward	5'-CATGTACGTTGCTATCCAGGC-3'
	Reverse	5'-CTCCTTAATGTCACGCACGAT-3'
β-Actin	Forward	5'-GGCTGTATTCCCCTCCATCG-3'
	Reverse	5'-CCAGTTGGTAACAATGCCATGT-3'

Table S4. Information of antibodies used in western blot analysis and flow metry analysis.

Antibodies	Company	Catalog number
Anti-L-Lactyl Lysine Rabbit mAb	PTM BIO	#PTM-1401RM
Anti-L-Lactyl-Histone H3 (Lys9)	PTM BIO	#PTM-1419RM
Aanti-L-Lactyl-Histone H3 (Lys18)	PTM BIO	#PTM-1406RM
Anti-L-Lactyl-Histone H4 (Lys8)	PTM BIO	#PTM-1415RM
Anti-L-Lactyl-Histone H4 (Lys12)	PTM BIO	#PTM-1411RM
Anti-Histone H3 Rabbit mAb (CT)	PTM BIO	#PTM- 1002RM
Anti-Histone H4 Rabbit mAb (CT)	PTM BIO	#PTM-1015RM

Anti-CXCL1/GRO alpha (mouse)	Abcam	#ab322200
Anti-CXCL1/GRO alpha (human)	Abcam	#ab89318
Anti-PDGFR alpha antibody	Abcam	#ab203491
β-ACTIN	CST	#4967S
Goat anti-mouse IgG-HRP	BOSTER	#BA1050
Goat anti-rabbit IgG-HRP	BOSTER	#BA1054
FITC anti-mouse CD14 Antibody	BioLegend	#123307
FITC anti-mouse Ly-6G Antibody	BioLegend	#127605

Table S5. Sequences of primers used in CUT&Tag-qPCR assay

Gene		Sequence
Cxcl1-A	Forward	5'-GAAACACCCTGTACTCCGGG-3'
	Reverse	5'-CTGGGATCATGGTGCTGTGT-3'
Cxcl1-B	Forward	5'-CTGCAGAGCCACACTCAAGA-3'
	Reverse	5'-ACCACAACTTTCCGACCTCC-3'
Cxcl1-control	Forward	5'-ACAGACTGCTCTGATGGCAC-3'
	Reverse	5'-CTCCCACACATGTCCTCACC-3'
