# Supplementary material for: Histone lactylation in fibroblast-like synoviocytes drives CXCL1-mediated immune cell recruitment in rheumatoid arthritis
Source: iScience. 2026 Nov 4;29(11):118108. doi: 10.1016/j.isci.2026.118108 (PMC13756642; doi:10.1016/j.isci.2026.118108)
Supplement: Supplementary file 2 [file mmc2.pdf]

Figure1 J

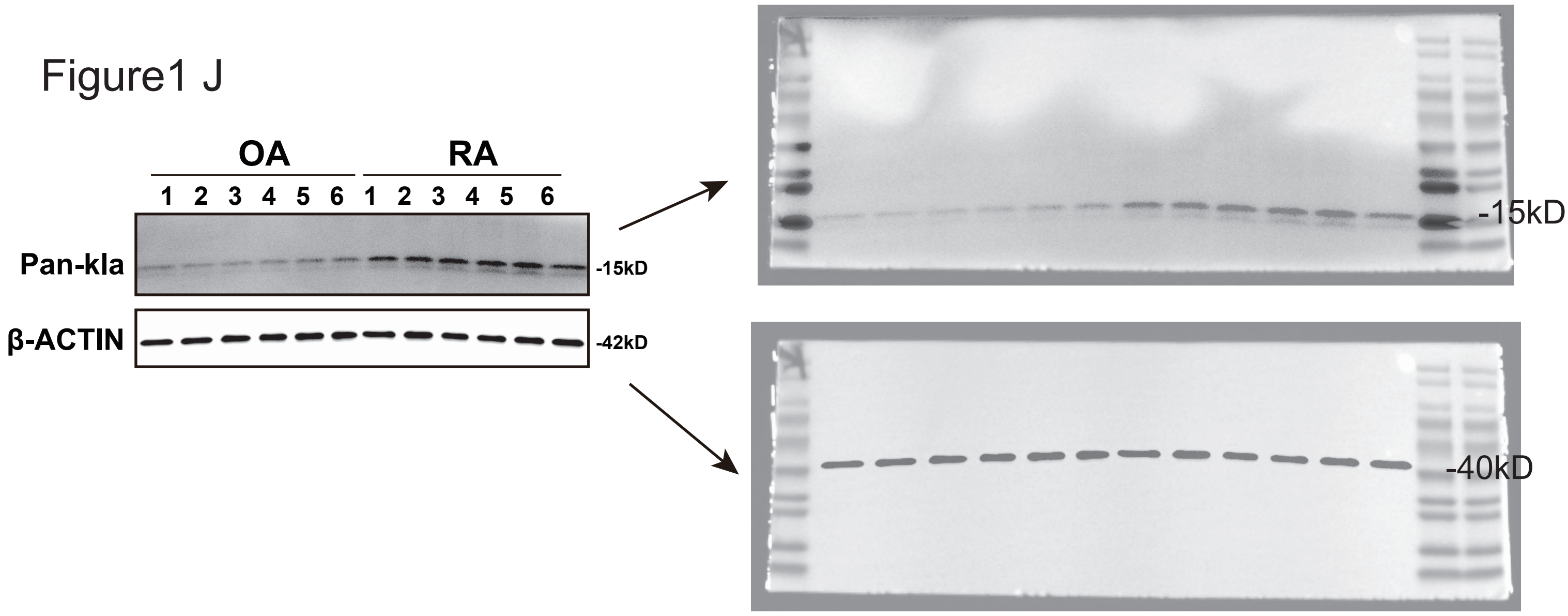

Figure1 K

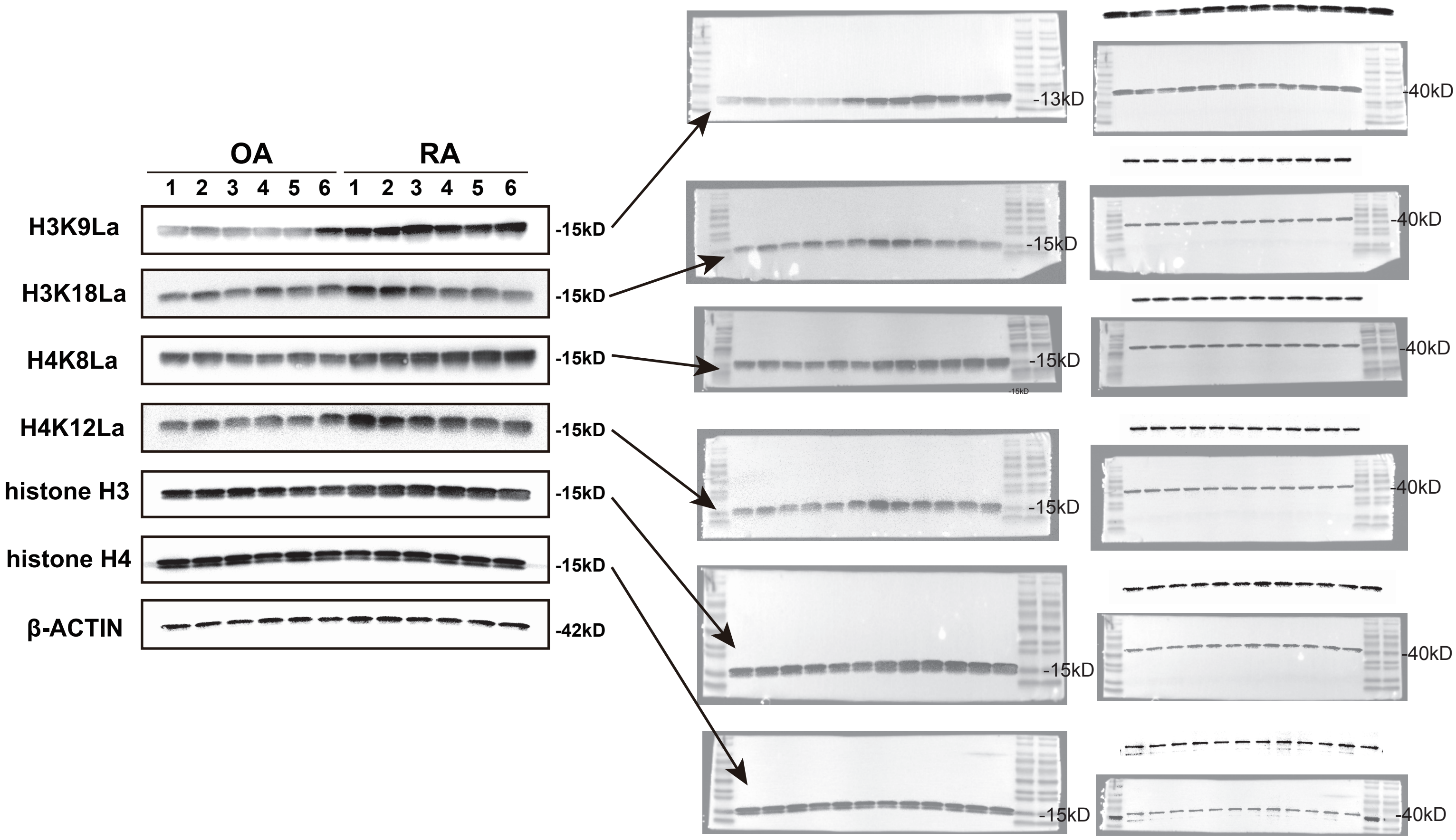

Figure2 B

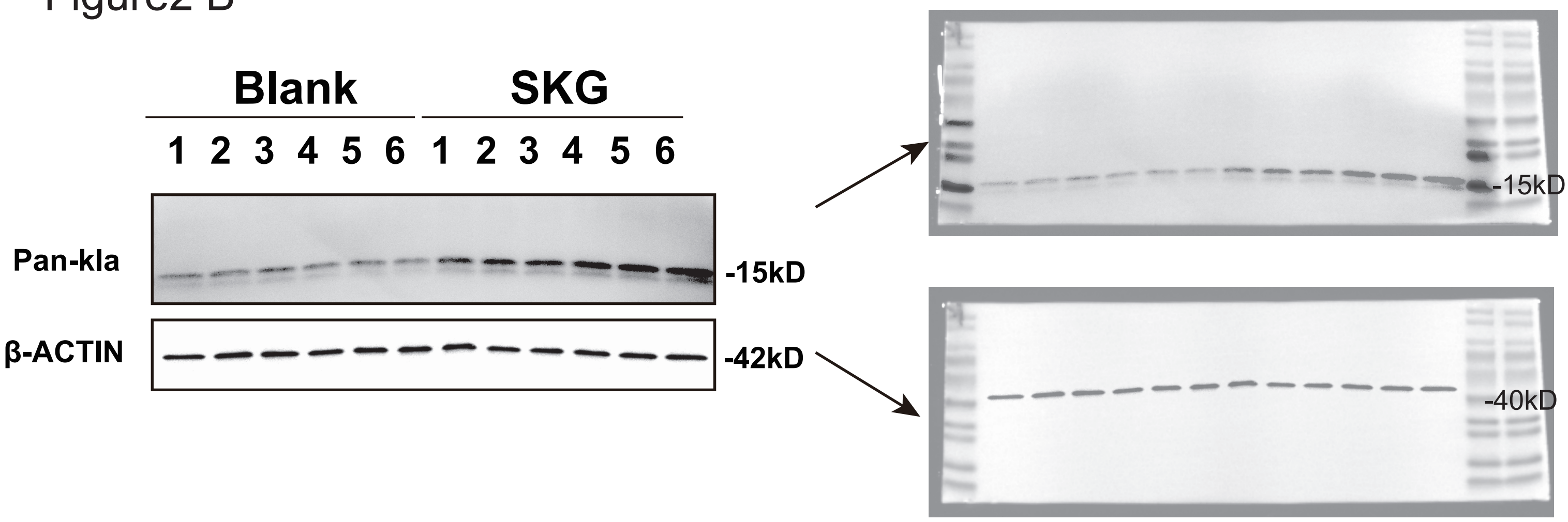

Figure2 F

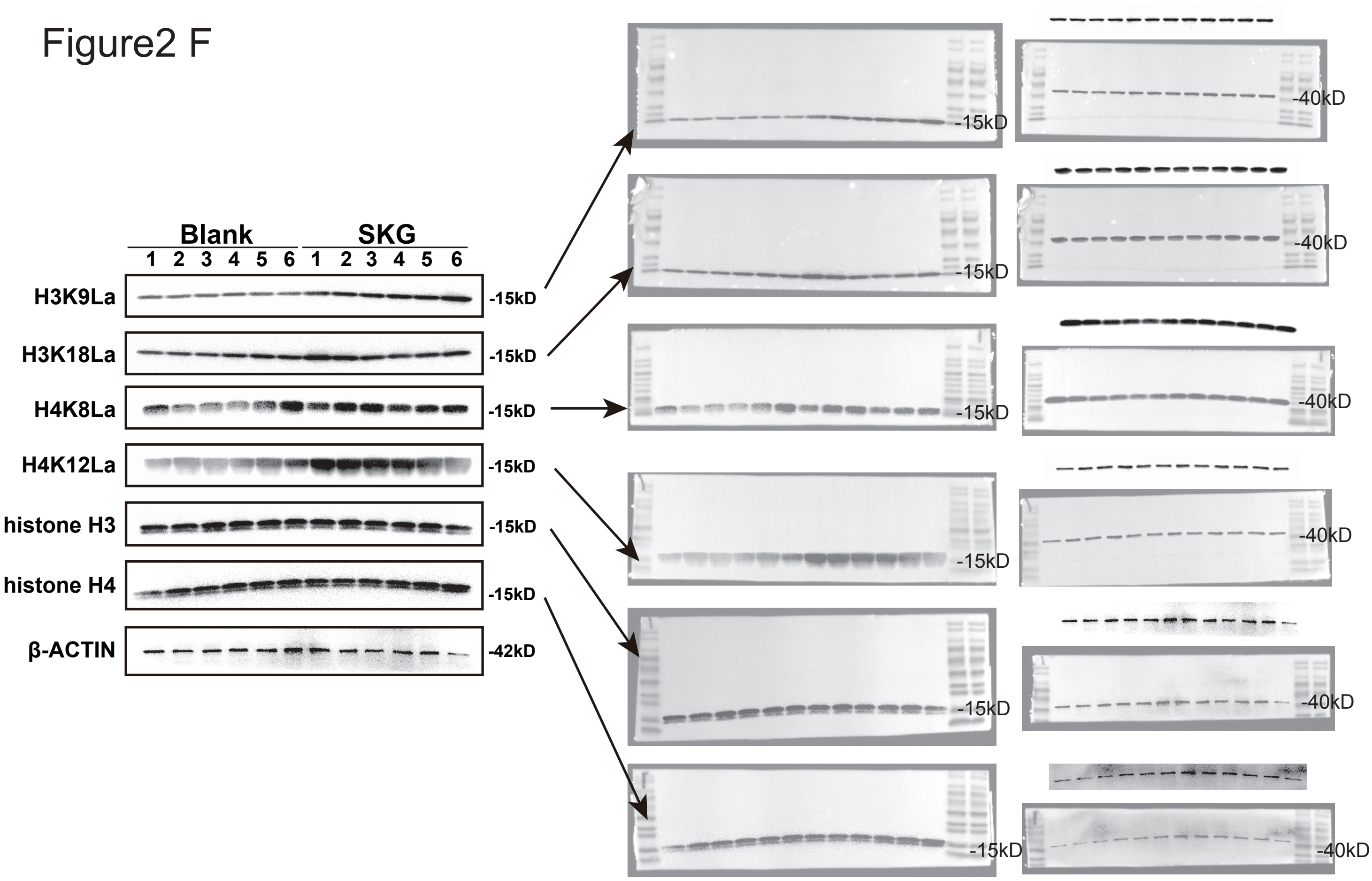

Figure2 C

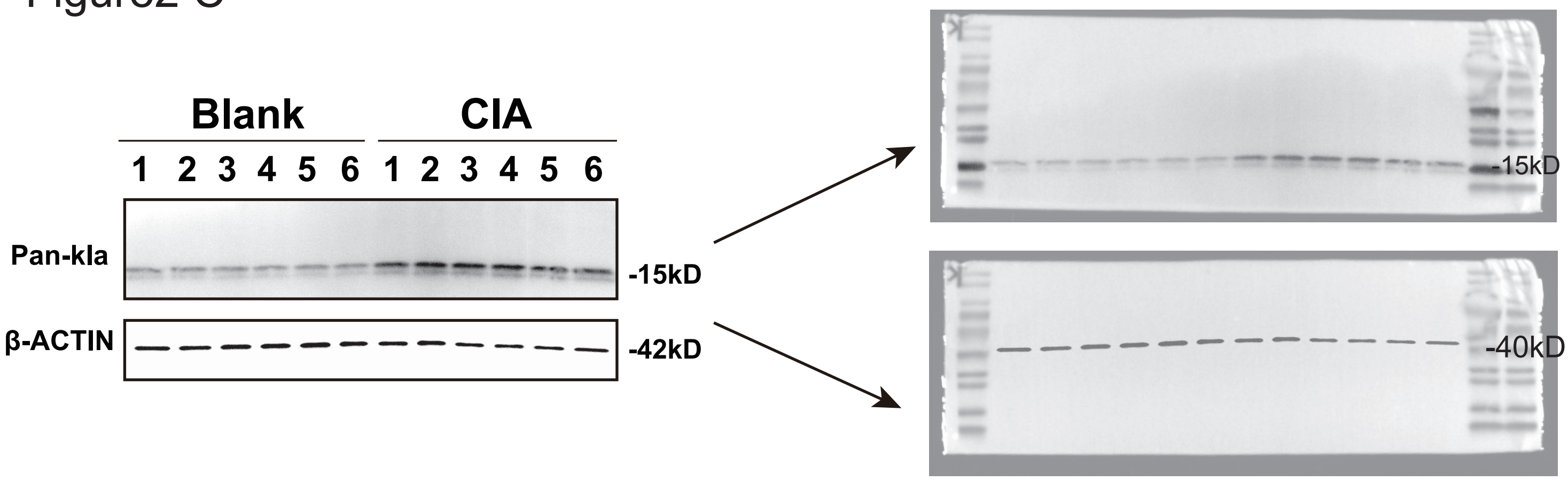

Figure2 G

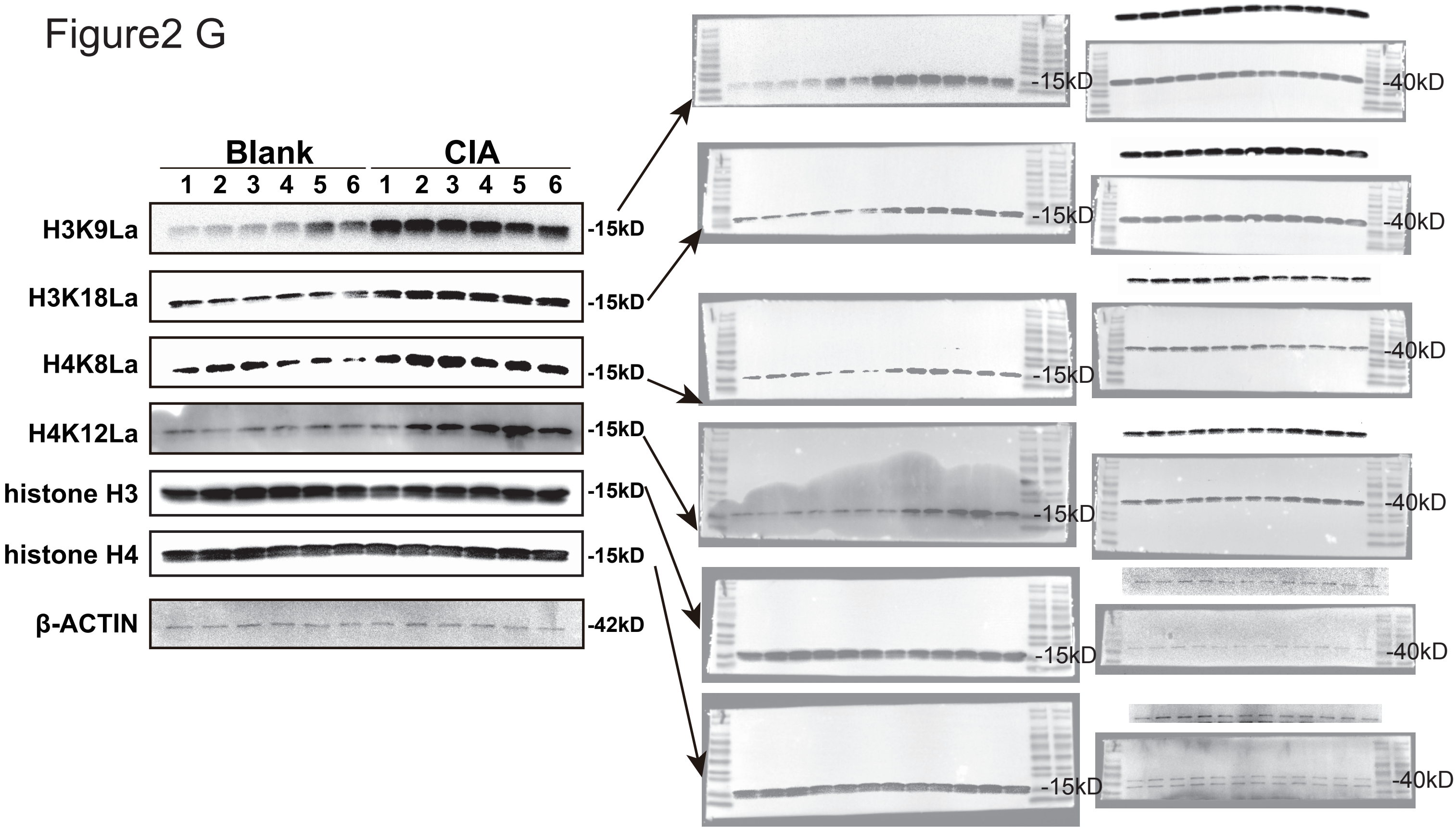

Figure3 A biological repeat 1

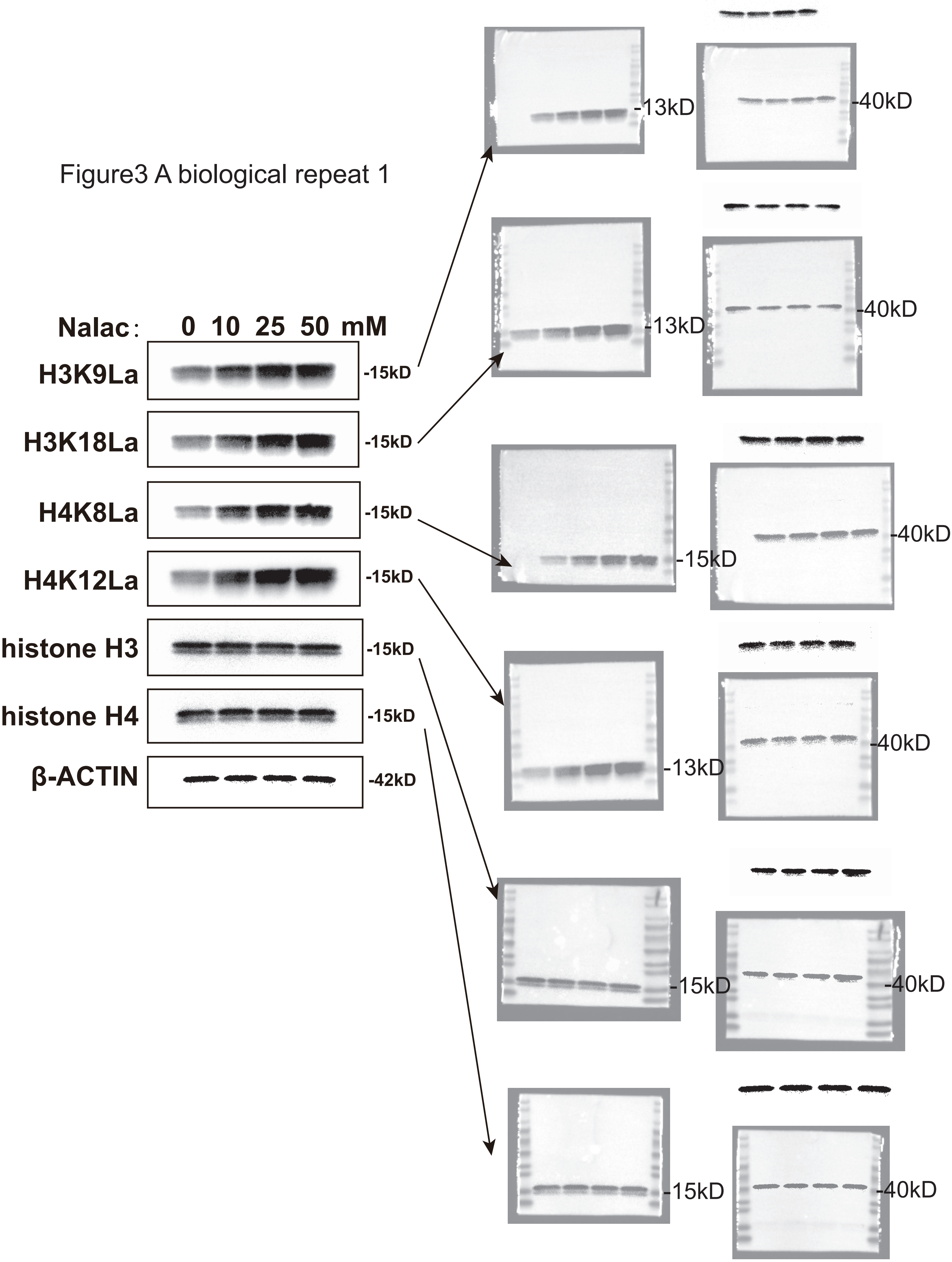

Figure3 A biological repeat 2

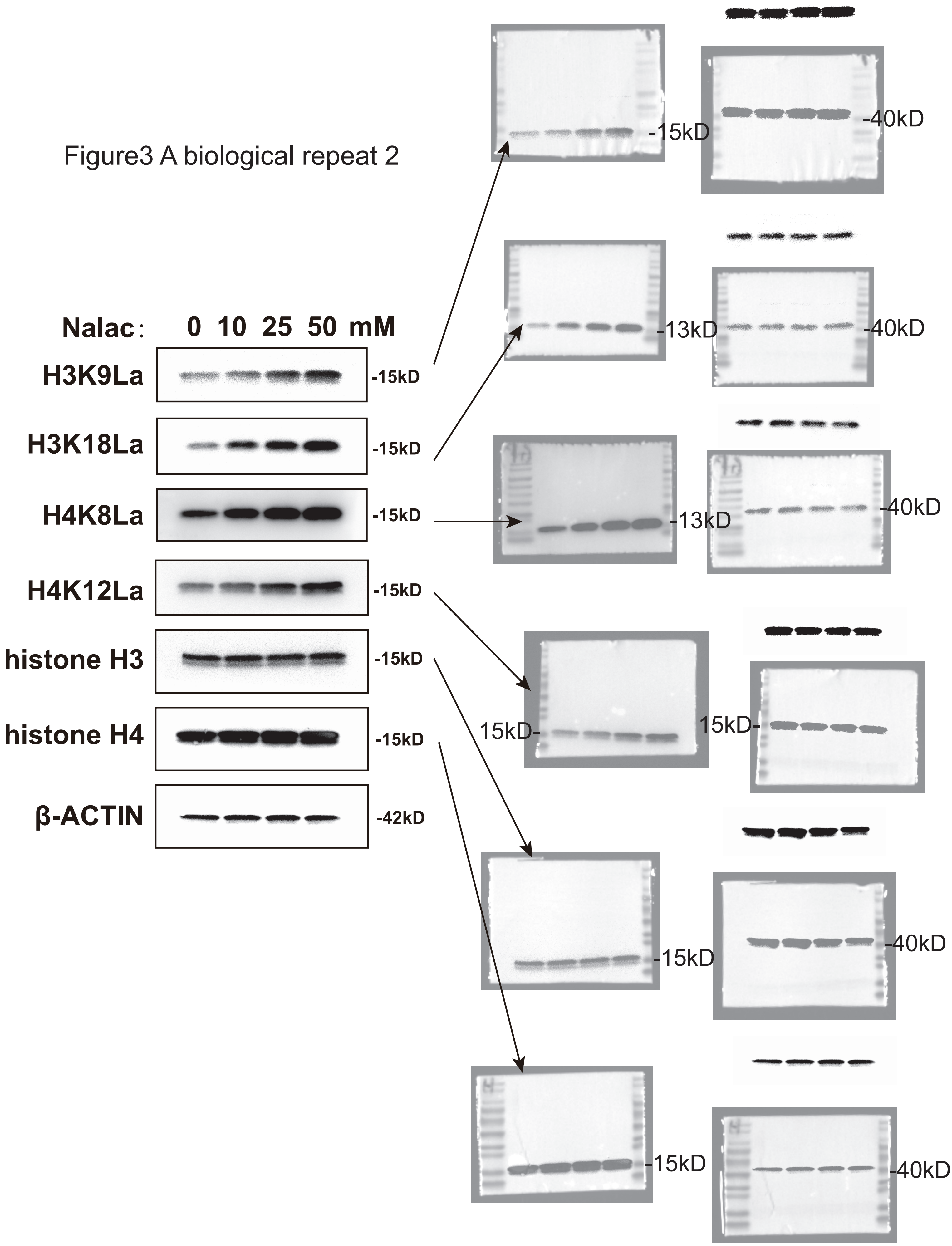

FigureS1A biological repeat 1

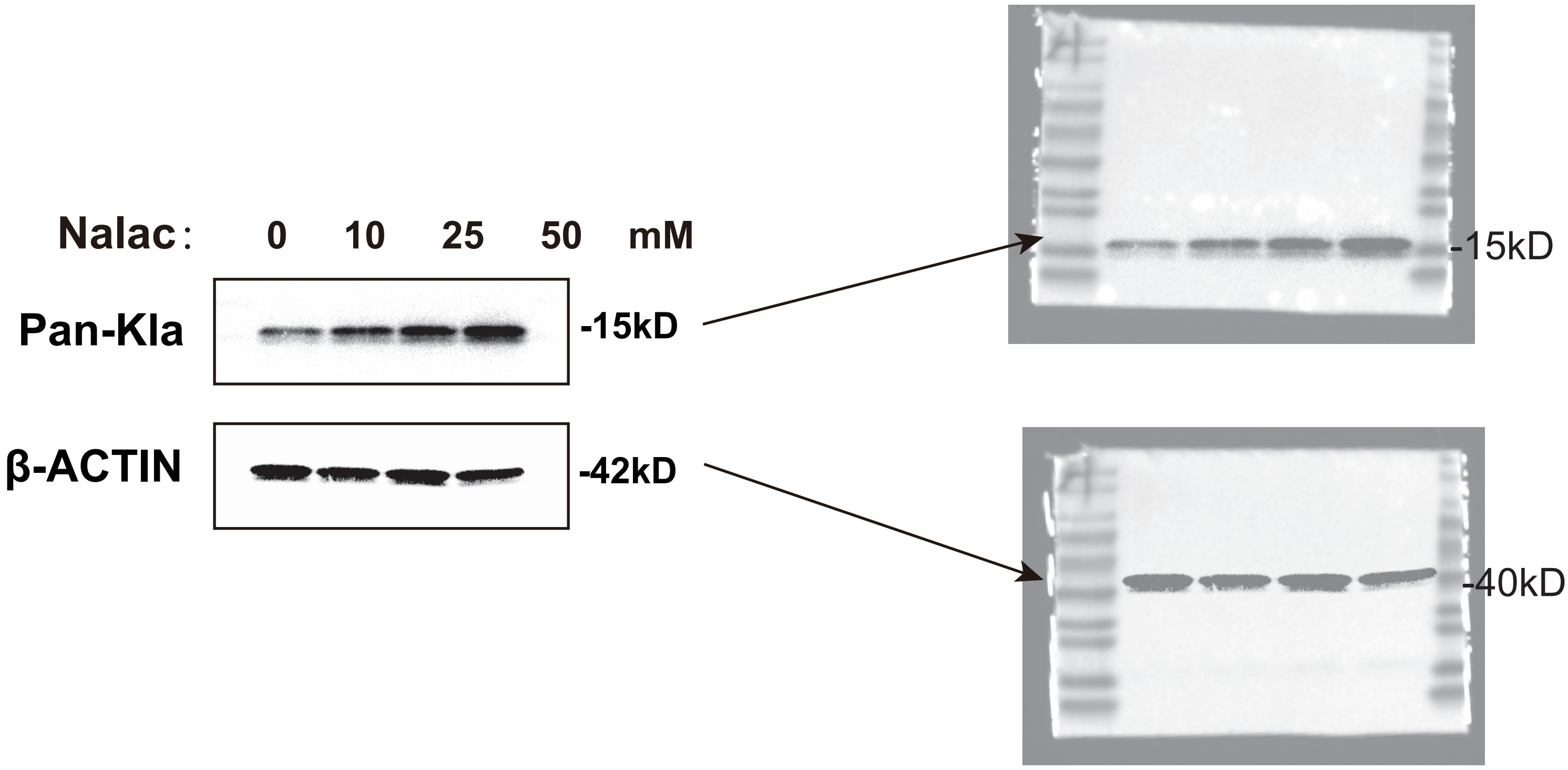

FigureS1B biological repeat 1

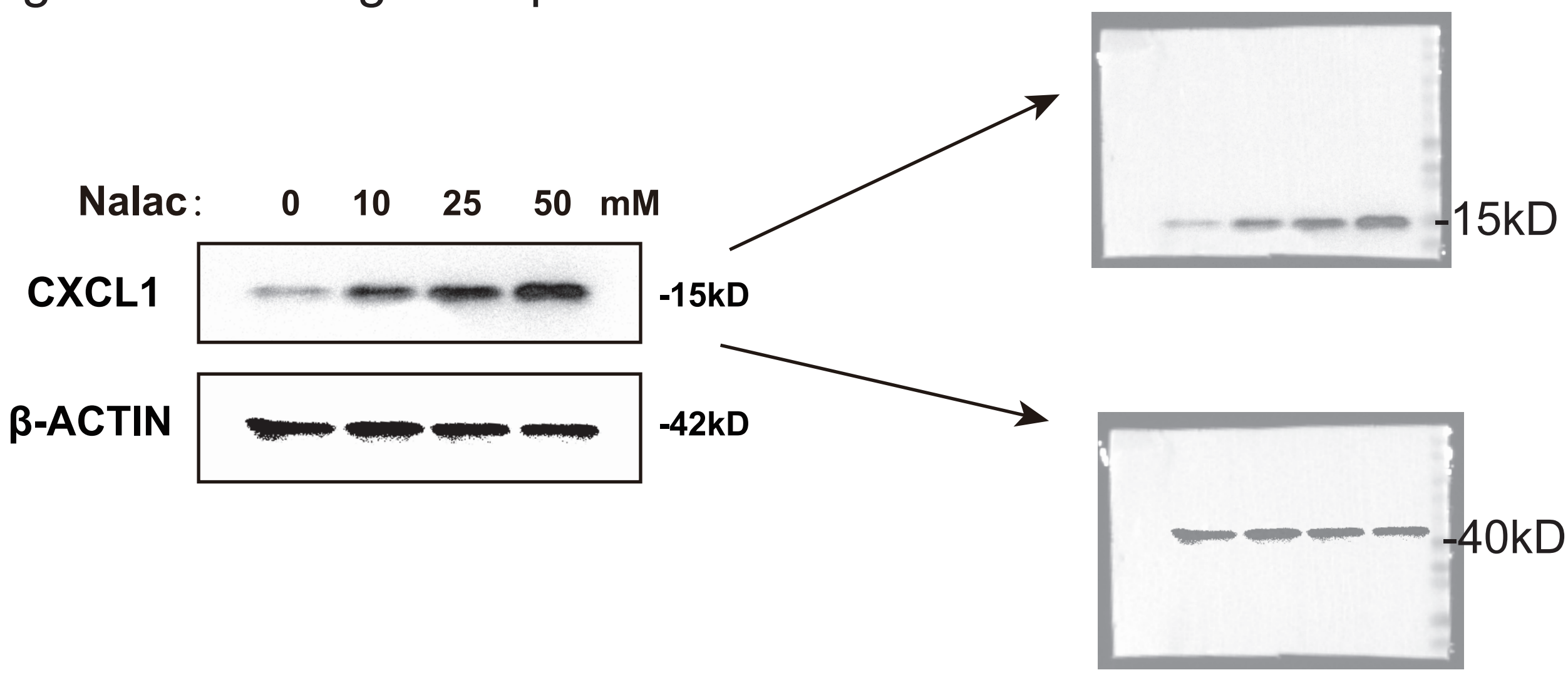

FigureS1A biological repeat 2

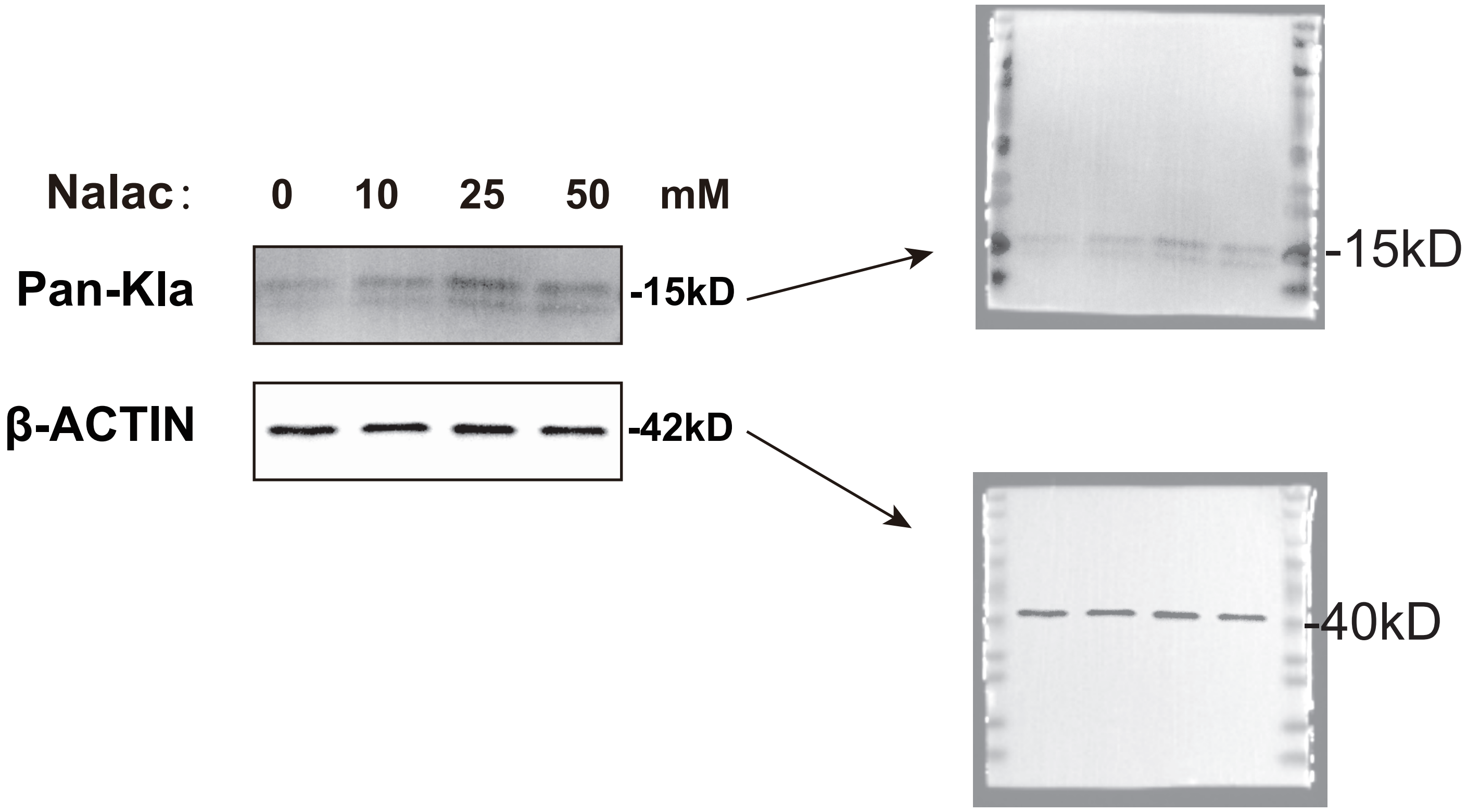

FigureS1B biological repeat 2

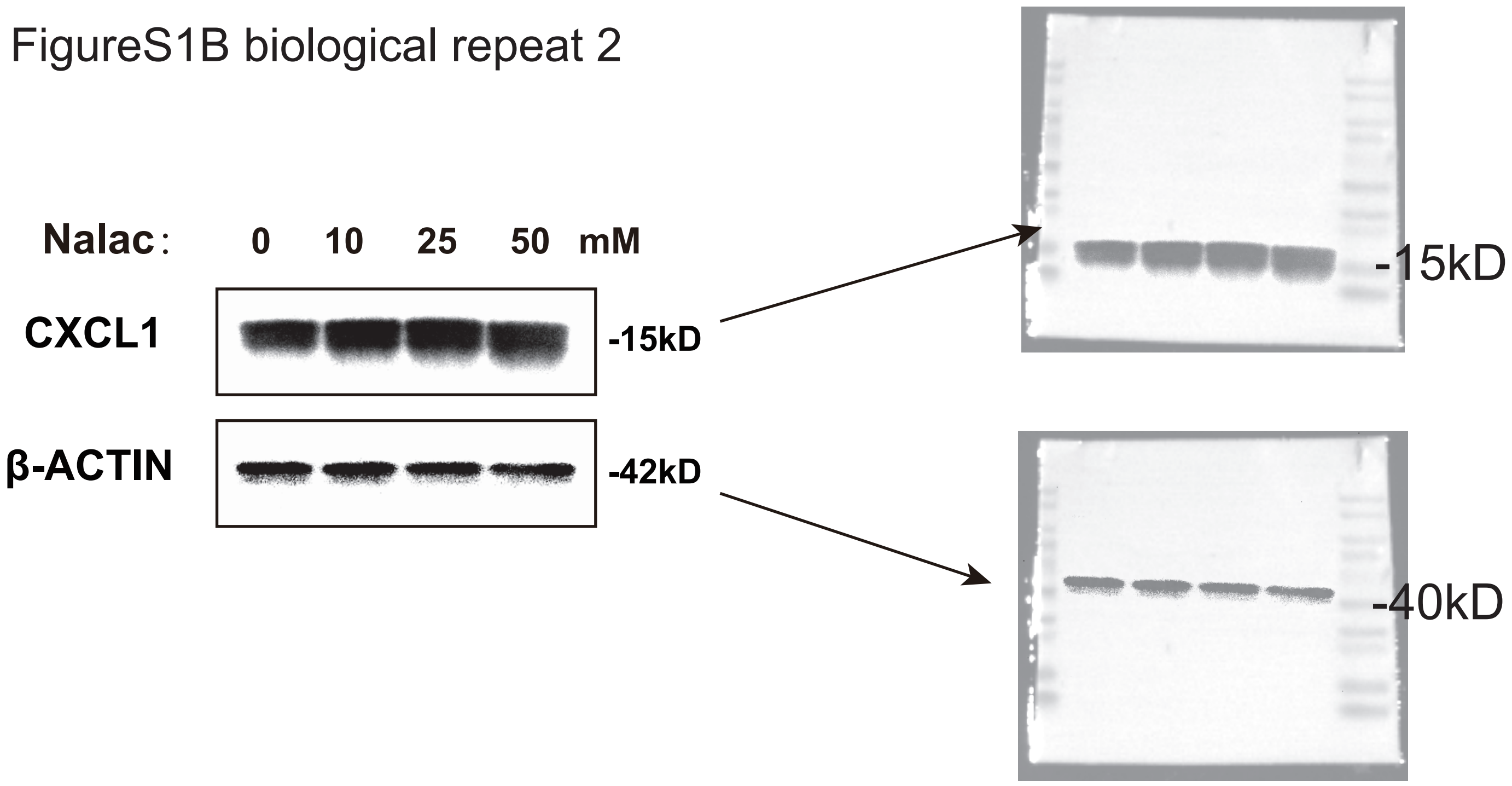

FigureS1C biological repeat 1

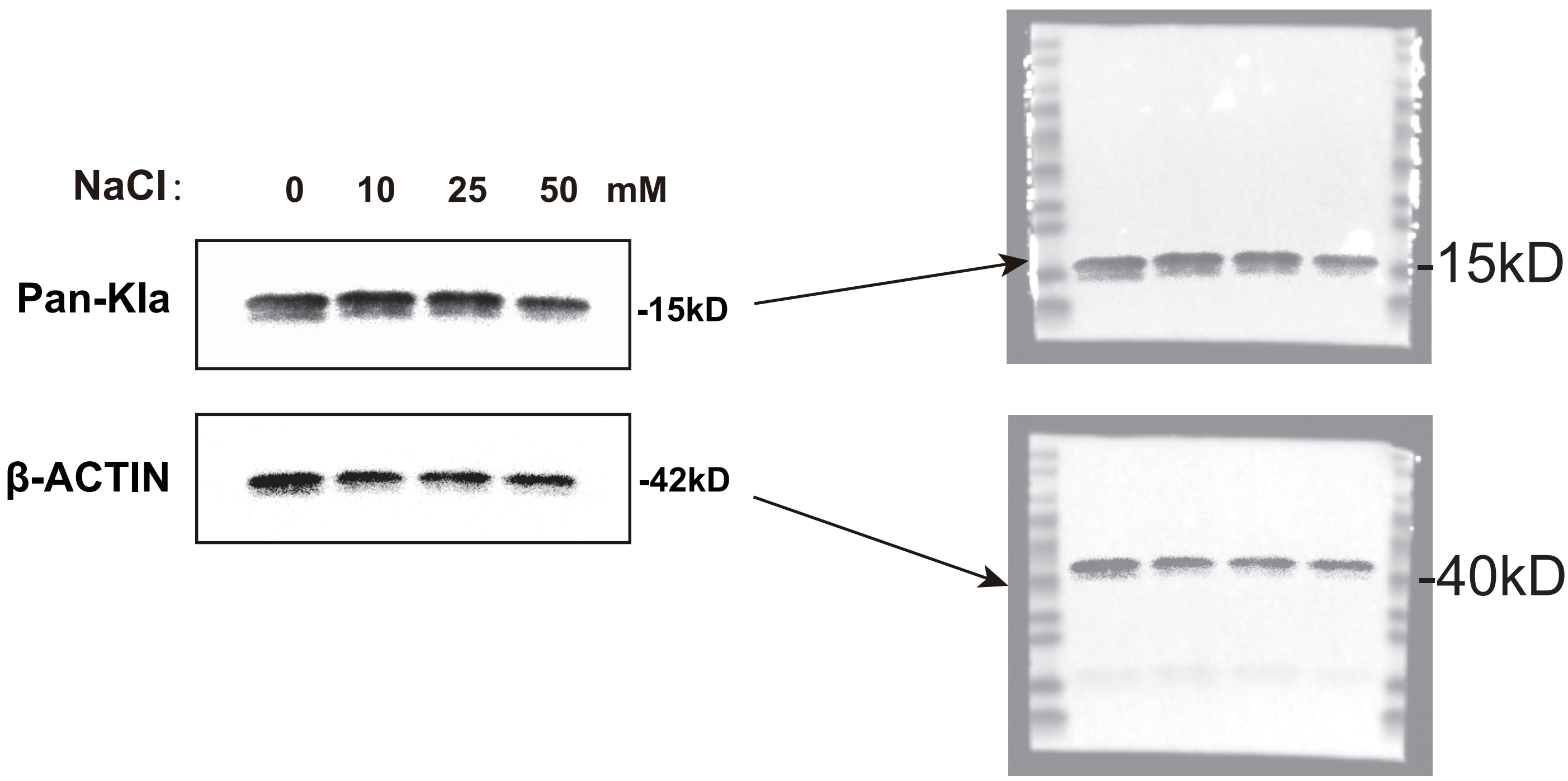

FigureS1D biological repeat 1

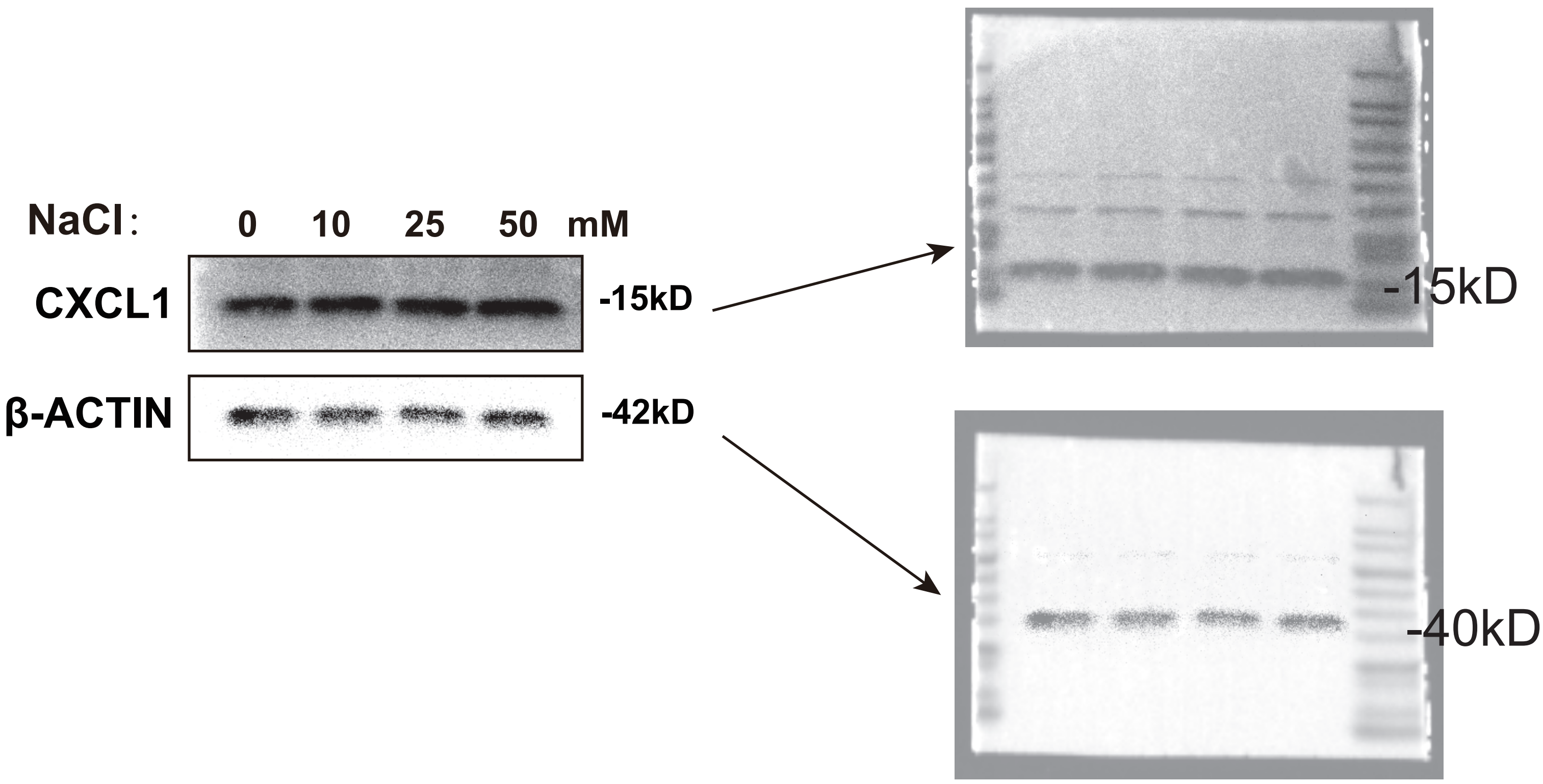

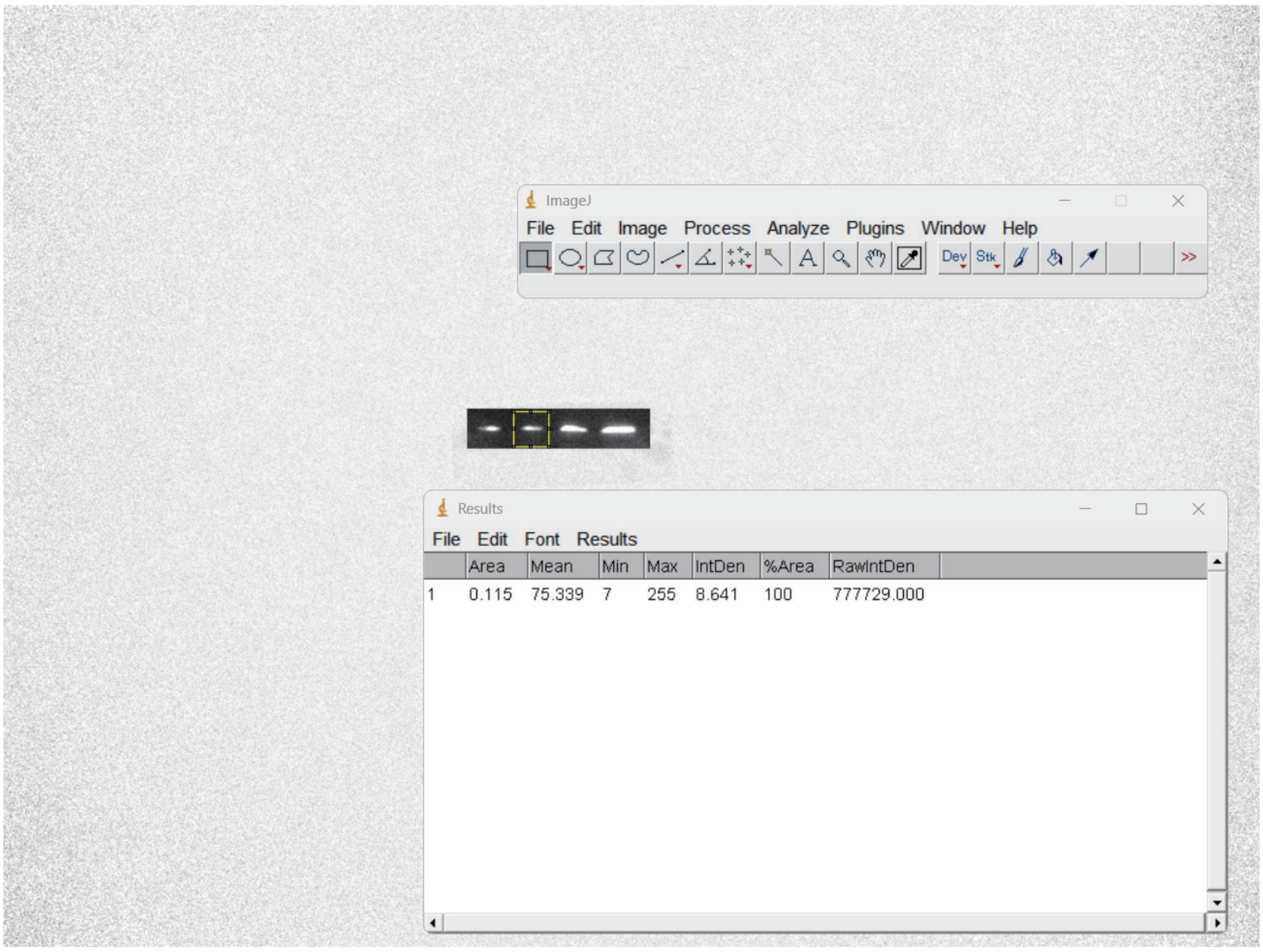

Western blot quantification

Proteins were resolved by SDS-PAGE and transferred onto PVDF membranes. Equal amounts of total protein (10 µg per lane) were loaded for all samples. After blocking, the membranes were incubated with primary antibodies against the target proteins, followed by the appropriate HRP-conjugated secondary antibodies, and chemiluminescent signals were detected using an ECL substrate and an imaging system.

Chemiluminescent images were acquired at increasing exposure times and exported as uncompressed 16-bit grayscale TIFF files. Quantification was performed only using an exposure in which the bands of interest and the adjacent background were within the linear detection range and no quantified pixels were saturated. Images used for quantitative analysis were not subjected to brightness or contrast adjustment, filtering, or other image processing. Where display adjustments were applied to duplicate copies for figure assembly, these adjustments were applied uniformly to the entire image and are disclosed in the corresponding figure legends.

After imaging the target protein, the same membrane was stripped and re-probed with an antibody against the corresponding loading control [β-actin]. Therefore, each target protein and its corresponding loading control were derived from the same gel, the same transferred membrane, and the same sample lanes. When the target protein and loading control required different exposure times, each signal was quantified using its own non-saturated exposure within the linear detection range. The target and loading-control measurements nevertheless corresponded to the same lanes of the same membrane.

Densitometric analysis was performed using ImageJ version 1.54 (National Institutes of Health, USA; Schneider et al., 2012). For each band, a rectangular region of interest of identical dimensions was placed over the band and over an adjacent signal-free region of the same membrane. The area, mean gray value, minimum and maximum gray values, and integrated density were recorded. Following local background subtraction, the background-corrected signal of each target band was normalized to the background-corrected signal of the corresponding loading-control band from the same lane of the same membrane. Where indicated, the resulting target-to-loading-control ratio was further expressed as fold change relative to the mean of the control group, which was set to 1.

Six independent biological replicates were analyzed for the relevant experiments ($n = 6$), unless otherwise specified in the figure legend. Data are presented as mean ± SD. Statistical comparisons were performed as specified in the corresponding figure legends. A two-sided p value of <0.05 was considered statistically significant.

For transparency, full-resolution, uncropped and unadjusted images of the target and loading-control detections are provided, including molecular-weight markers, complete sample lanes and exposure information. Each target image is linked to the corresponding loading-control image from the same membrane through a unique blot identifier. No intensity-based processing other than the local background subtraction described above was used for quantitative analysis.

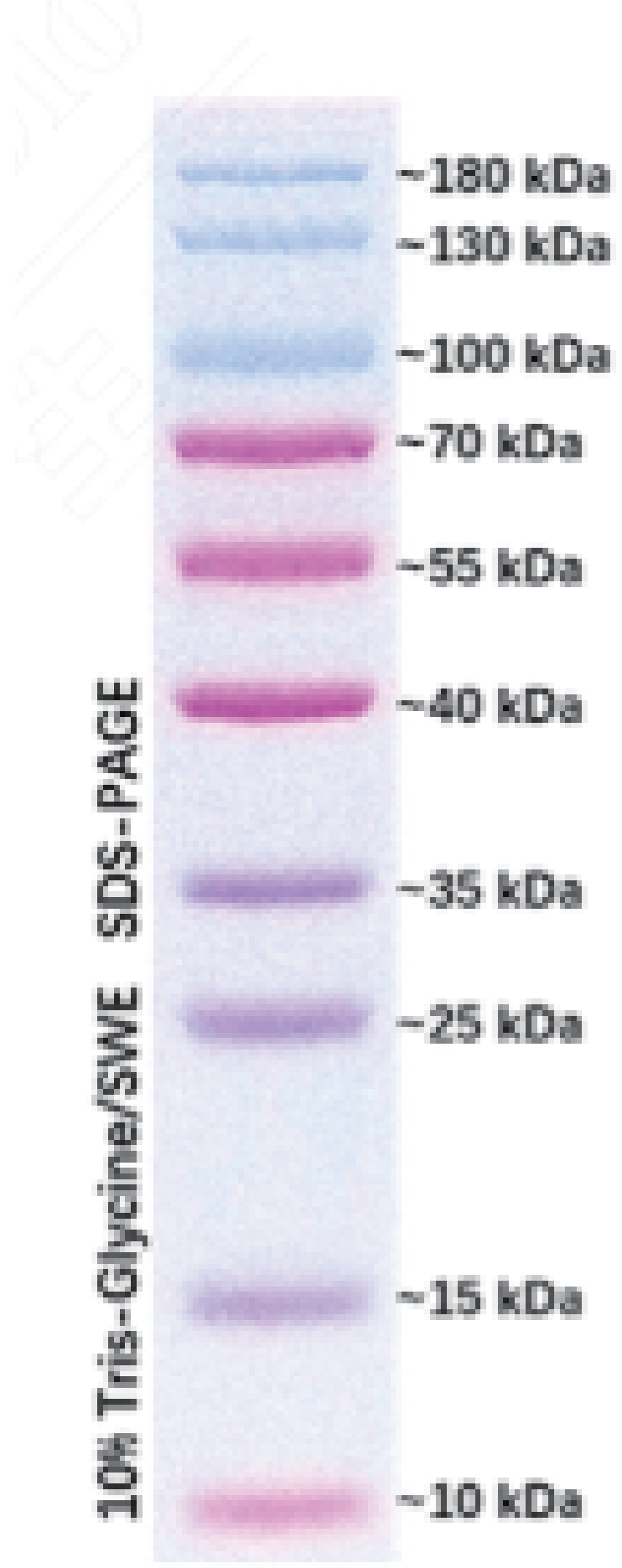
~180 kDa
~130 kDa
~100 kDa
~70 kDa
~55 kDa
~40 kDa
~35 kDa
~25 kDa
~15 kDa
~10 kDa
10% Tris-Glycine/SWE SDS-PAGE

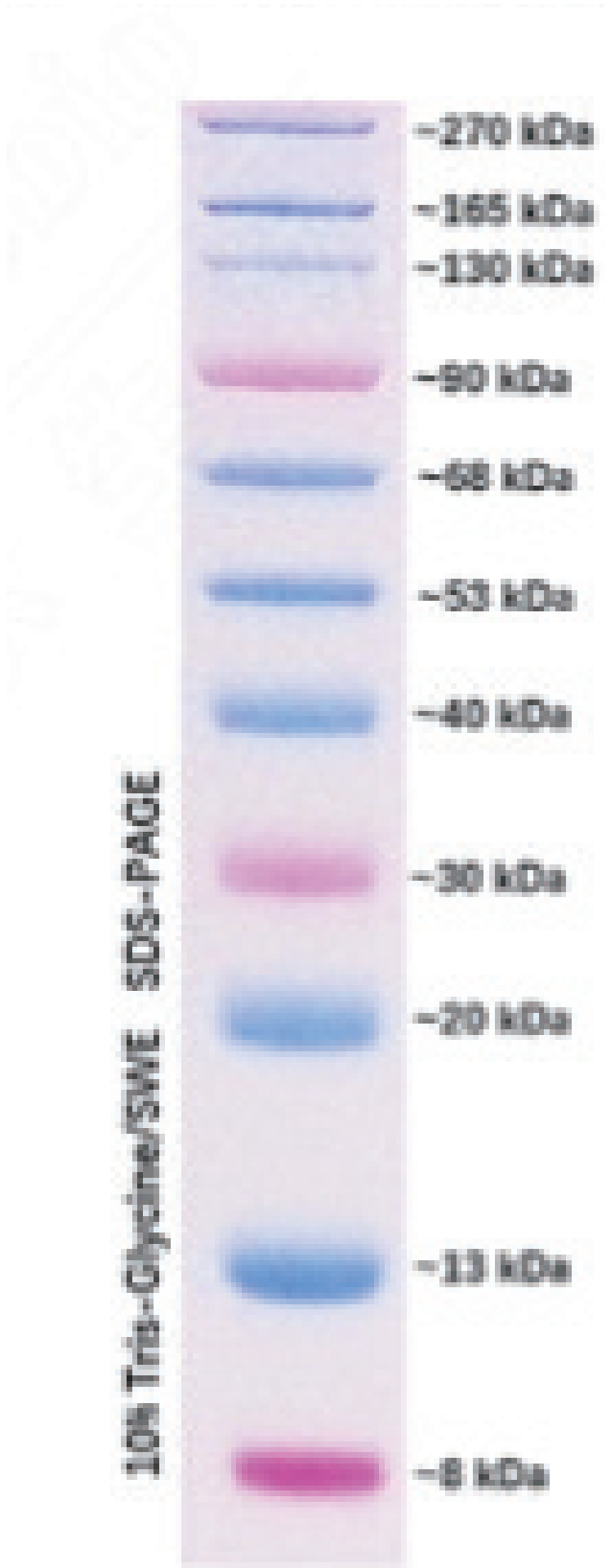
~270 kDa
~165 kDa
~130 kDa
~90 kDa
~68 kDa
~53 kDa
~40 kDa
~30 kDa
~20 kDa
~13 kDa
~8 kDa
10% Tris-Glycine/SWE SDS-PAGE
